# Impacts of Differentially Shaped Silver Nanoparticles with Increasingly Complex Hydrophobic Thiol Surface Coatings in Small-Scale Laboratory Microcosms

**DOI:** 10.3390/nano14080654

**Published:** 2024-04-09

**Authors:** Bryan J. Harper, Arek M. Engstrom, Stacey L. Harper, Marilyn R. Mackiewicz

**Affiliations:** 1 Department of Environmental and Molecular Toxicology, Oregon State University, Corvallis, OR 97331, USA; bryan.harper@oregonstate.edu (B.J.H.); arek.engstrom@gmail.com (A.M.E.); 2 School of Chemical, Biological, and Environmental Engineering, Oregon State University, Corvallis, OR 97331, USA; 3 Oregon Nanoscience and Microtechnologies Institute, Corvallis, OR 97331, USA; 4 Department of Chemistry, Oregon State University, Corvallis, OR 97331, USA

**Keywords:** microcosm, *Daphnia*, algae, bacteria, zebrafish, lipid membranes, silver ion release, surface oxidation, lipid nanoparticles

## Abstract

We investigated the impacts of spherical and triangular-plate-shaped lipid-coated silver nanoparticles (AgNPs) designed to prevent surface oxidation and silver ion (Ag^+^) dissolution in a small-scale microcosm to examine the role of shape and surface functionalization on biological interactions. Exposures were conducted in microcosms consisting of algae, bacteria, crustaceans, and fish embryos. Each microcosm was exposed to one of five surface chemistries within each shape profile (at 0, 0.1, or 0.5 mg Ag/L) to investigate the role of shape and surface composition on organismal uptake and toxicity. The hybrid lipid-coated AgNPs did not result in any significant release of Ag^+^ and had the most significant toxicity to *D. magna*, the most sensitive species, although the bacterial population growth rate was reduced in all exposures. Despite AgNPs resulting in increasing algal growth over the experiment, we found no correlation between algal growth and the survival of *D. magna,* suggesting that the impacts of the AgNPs on bacterial survival influenced algal growth rates. No significant impacts on zebrafish embryos were noted in any exposure. Our results demonstrate that the size, shape, and surface chemistry of AgNPs can be engineered to achieve specific goals while mitigating nanoparticle risks.

## 1. Introduction

Understanding how the physicochemical features (size, shape, surface chemistry, and charge) of silver nanoparticles (AgNPs) influence their behavior in biological systems is critical for developing novel and biocompatible engineered AgNPs. They are highly desired because of their unique optoelectronic properties that are governed by their size, shape, oxidative state, and surface chemistry [1,2,3,4]. Although commonly used because of their excellent antimicrobial properties [5,6,7,8,9,10,11], AgNPs are developed as catalysts [12,13,14,15], conductive inks [16,17,18], X-ray imaging agents [19,20], photothermal agents [21,22,23], and drug delivery vehicles [24,25]. Given that around 500 tons of AgNPs are produced annually for industrial and biomedical applications, they will inevitably be released into aquatic environments, raising concerns about their ecosystem risks [26,27,28].

Most AgNPs release Ag^+^ ions due to surface oxidation, complicating understanding of their potential risks to aquatic systems [29]. These Ag^+^ ions then interact with sulfides [30], humic compounds [31], chlorides [30], and other species to form compounds that confound our understanding of nanoparticle-induced toxicity [32]. Additionally, toxicity testing with individual species lacks the environmental diversity of community interactions impacted by toxicants, making understanding their ecological risk difficult [33]. To begin to address this, mesocosm experiments are often performed, but these studies typically require large amounts of test materials due to their size, generate much waste, and are prohibitive in cost, time, and resources [34]. Thus, alternative small-scale testing strategies employing multiple species from differing trophic levels are one way to close the data gap for nanomaterials [35,36,37]. These types of studies have proven beneficial in understanding the fate and toxicity of nanomaterials, showing how community complexity can mitigate the toxicity to some organisms [36].

To overcome the challenge posed by the presence of Ag^+^ ions found in AgNP toxicity testing, Mackiewicz et al. synthesized a unique set of AgNPs to address critical data gaps for environmental risk evaluation of AgNPs [38,39,40]. These efforts also aimed to pave the way for developing novel and biocompatible engineered nanomaterials. Furthermore, this hybrid lipid-coating membrane is an adaptable architecture that “shape-locks” the AgNPs to minimize changes in shape or size that may affect their unique optoelectronic properties or confound ecotoxicity studies [38]. These AgNPs were synthesized by carefully selecting lipid membrane coatings with altered surface chemistries designed to minimize the dissolution of Ag^+^ ions and determine the most suitable surface chemistries for biological applications [38]. Early studies with these nanoparticles have shown that size is a dominant factor in toxicity, followed by shape in vivo with embryonic zebrafish [39,40]. Thus far, only single-species experiments have been performed with these robust AgNPs shielded from surface oxidation. That is, microcosm-style experiments involving multiple species to understand the role of nanoparticle surface modifications in community exposures and their fate are still needed.

The increased chemical engineering knowledge concerning AgNP synthesis has allowed us to synthesize AgNPs of various shapes [41] with diverse surface chemistries [42]. It is possible to apply similar surface chemistries to spherical AgNPs (AgNSs) and triangular-plate-shaped AgNPs (AgNPLs) of comparable sizes using established synthesis methods. The comparison between the two nanoparticle shapes is intriguing because the impact of surface chemistry on Ag^+^ ion dissolution may differ due to the geometry of triangular AgNPs as opposed to the surfaces of AgNSs. Furthermore, as the size of these nanoparticles is incredibly small and biologically significant [43], it is likely that the shape of the AgNPs may affect their uptake by organisms or their interaction with their membranes [44]. By studying the uptake and effects of the AgNPs of different shapes and with various surface chemical functionalities, valuable information about the environmental risks associated with engineered AgNPs can be gained. For example, assuming that the edges and tips of the AgNPLs are less able to be stabilized against Ag^+^ ion dissolution due to the attached surface chemistry than the AgNSs, this would result in increased dissolution, which may impact those species more susceptible to Ag^+^ than those who may ingest the nanoparticle itself. In addition, membranes of bacterial or algal cells may differentially interact with nanoparticles with sharper edges rather than those that are more spherical.

**Scheme 1:**
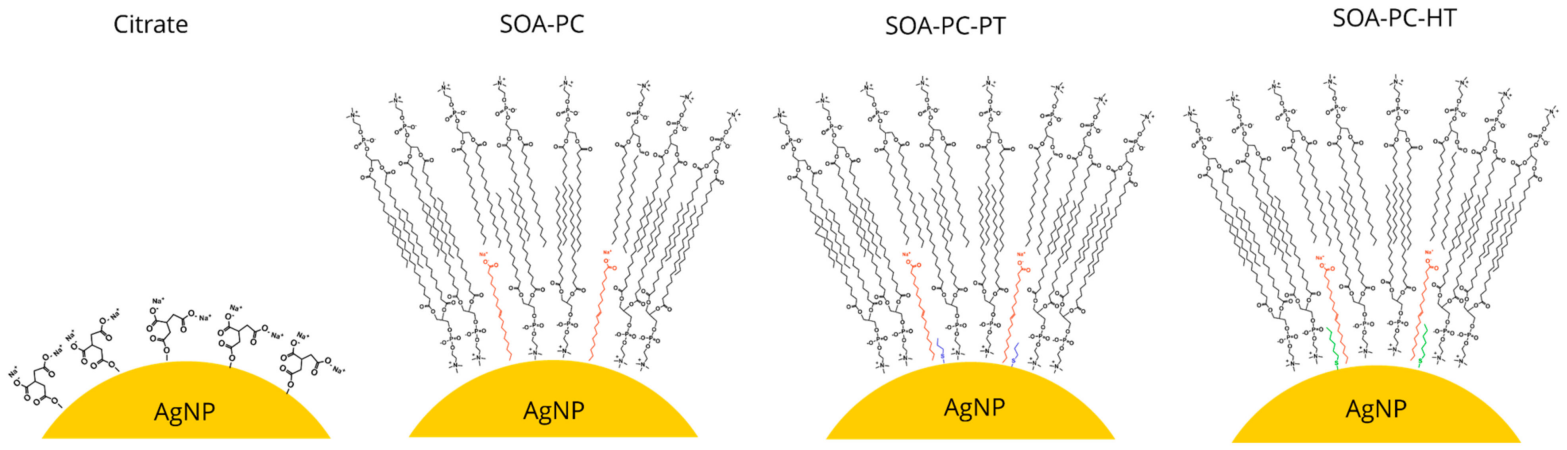
Schematic representation of the particle surface chemistry on the engineered AgNPs.

Simple ionic surface stabilization of AgNPs can be achieved easily with chemicals such as citrate; however, these undergo surface oxidation and Ag^+^ ion release over time [45]. However, steric stabilization of AgNPs that minimizes oxidative dissolution with hybrid lipid structures has received little attention, especially in systems with multiple species [38,39,40]. In this study, we sought to examine how shape, surface chemistry, and Ag^+^ ion release from surface oxidation impacts a diverse community of organisms using a microcosm model. Citrate-capped AgNSs and AgNPLs were synthesized and chemically engineered with a sequentially more complex hybrid lipid membrane coating mainly composed of L-α-phosphatidylcholine (PC) lipids (Scheme 1). The coatings include membrane architectures composed of sodium oleate and PC (SOA-PC) and those in which either a short-chained thiol, propanethiol (SOA-PC-PT), or long-chained thiol, hexanethiol (SOA-PC-HT) was used to anchor and stabilize the membrane architecture in a tight packing arrange to stabilize the AgNPs (Scheme 1). Beyond the impacts of particle shape and diverse surface chemistries, there is a need to understand the importance of various synthesis methods where the purity of the nanomaterials may impact toxicity to the organisms [46]. Therefore, the most complex and theoretically stable surface chemistry (SOA-PC-HT) was subjected to additional purification steps to evaluate the overall stability of the nanoparticle and determine if the impacts on organisms are significantly altered by the extra purification step.

Some may argue that AgNPs exist naturally in the environment and thus provide little risk at environmentally relevant concentrations. While this may be true, it may also be true that engineered surfaces or environmentally acquired coronas, as well as different engineered shapes, may pose differing risks relative to naturally occurring AgNPs. In addition, the risks in terms of the most sensitive species impacted by the nanoparticle exposure must also be considered. While it is often difficult to accurately simulate the natural world, our test design allows for a broader ecological understanding which enables us to better evaluate the impacts on sensitive species and extrapolate risks to larger real-world systems.

## 2. Materials and Methods

### 2.1. Reagents for Nanoparticle Synthesis

Trisodium citrate dihydrate (Cit) 99% (Na_3_C_6_H_3_O_7_), 1-hexanethiol (HT) 95% (CH_3_(CH_2_)_4_CH_2_SH), 1-propanethiol (PT) 99% (CH_3_CH_2_CH_2_SH) and chloroform were purchased from Sigma Aldrich (St. Louis, MO, USA). Silver nitrate 98% (AgNO_3_) was purchased from G. Frederick Smith Chemical Company (GFS) (Powell, OH, USA). Hydrogen peroxide 30% (H_2_O_2_), HEPES, and sodium borohydride 99% (NaBH_4_) were obtained from VWR Chemicals (Radnor, PA, USA). L-α-phosphatidylcholine was purchased from Avanti Lipids Alabaster, AL, USA). Sodium Oleate (SOA) 97% was obtained from Tokyo Chemical Industry Co, Ltd. (Portland, OR, USA). Ten mM sodium phosphate buffer was prepared for PC suspension at pH 8. Instant Ocean Salts were purchased from Aquatic Ecosystems (Forth Collins, CO, USA). Nanopure water was obtained from a Milli-Q ultrapure system. All chemicals were used as received.

### 2.2. UV-Vis, Dynamic Light Scattering (DLS) and Zeta Potential Measurements (ZP)

Absorbance measurements were recorded in deionized water using a Agilent Cary 5000 UV-Vis-NIR spectrophotometer (Santa Clara, CA, USA) with a 1.0 cm path length quartz cell cuvette. Hydrodynamic diameter (HDD) and ZP were measured by DLS using a Malvern Zetasizer Nano ZS (Malvern Instruments, Worcestershire, UK) at a concentration of 0.5 mg Ag/L in the experimental media listed below. All measurements were conducted in triplicate.

### 2.3. Nanoparticle Synthesis and Characterization

**Figure 1:**
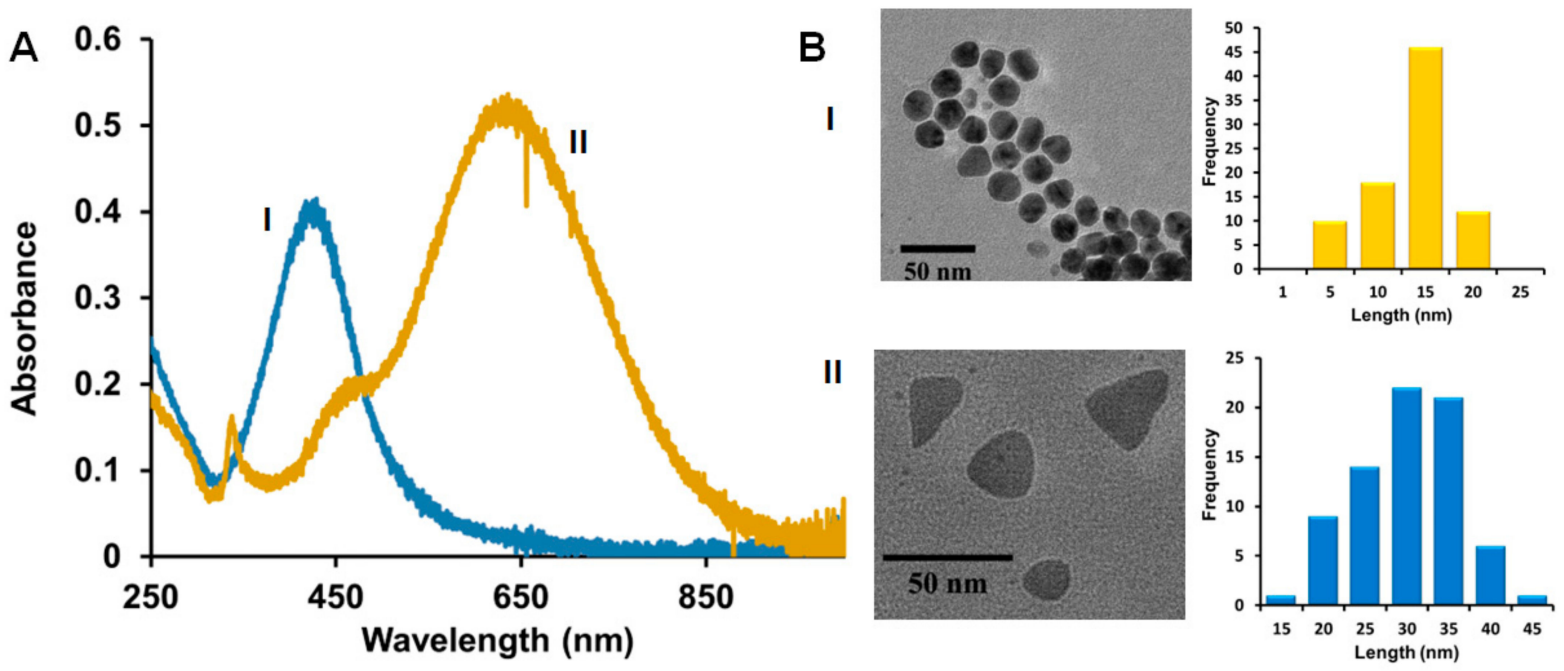
(**A**) Representative UV-Vis spectra; (**B**) Representative TEM distribution and histogram analysis of hybrid lipid-coated (I) AgNSs and (II) AgNPLs. TEM scale bar is 50 nm.

The starting Cit-capped spherical and triangular-plate AgNPs were prepared using a modified procedure described previously before applying the other coatings [39,47]. The AgNPs with SOA-PC, SOA-PC-PT, and SOA-PC-HT were prepared as described previously [38,39]. Briefly, freshly prepared spherical and triangular-shaped Cit-capped AgNPs were diluted to a final maximum optical density (O.D.) of 1.2 and 0.8 respectively. To form the Ag-SOA-PC nanoparticles, SOA (1.1 μL, 9.4 mM in H_2_O) was added to 1 mL of Cit-capped AgNPs placed in a vial. The Ag-SOA solution was vortexed for 5 sec and allowed to incubate for 20 min. This was followed by the addition of PC liposomes (10.4 µL, 0.32 mM in 10 mM sodium phosphate buffer at pH 8) that were vortexed for 5 sec and incubated for 40 min to form Ag-SOA-PC. To form the Ag-SOA-PC-PT and Ag-SOA-PC-HT, 1.4 μL of thiol (PT or HT at 30 mM in ethanol) was added to 1 mL of Ag-SOA-PC. The resulting Ag-SOA-PC-thiol (PT or HT) solution was vortexed for 5 sec and allowed to incubate at room temperature for at least 30 min before use. The Ag-SOA-PC-HT nanoparticles were further purified by incubating the sample with 10 mM Tween20 (20 μL per mL of AgNPs) to remove “nanoparticle-free” liposomes, followed by ultracentrifugation (4700 rpm for 4 min) using a Vivaspin column with a MWCO of 10 kDa. The AgNPs were washed 6 times with 15 mL of H_2_O and resuspended back to the same starting volume to yield Ag-SOA-PC-HT (P). Both spherical and triangular AgNPs were prepared with the following compositions: Cit, SOA-PC, SOA-PC-PT, SOA-PC-HT, and SOA-PC-HT (P). To determine if each batch of Ag-SOA-PC-HT was shielded from oxidation, a well-known cyanide (CN^-^) etch test was performed [48]. Briefly, an 800 µL solution of AgNPs with O.D. of 0.4 in H_2_O was incubated with 20 µL of 307 mM KCN for 1 h. The UV-Vis spectra were collected before and after the addition of KCN and the change in the O.D. and λ_max_ monitored. AgNPs are similar to those previously characterized by UV-Vis spectroscopy and TEM (see Figure 1 and Figure S1) [38,39].

### 2.4. Exposure Nanocosm Media (NCM)

Exposure media for the microcosm experiments was prepared as 50% by volume Taub’s #36 solution [49] with 50% solution of 5 mM HEPES buffer (EMD Chemicals Inc., Gibbstown, NJ, USA) adjusted to pH 7.2 using 0.1 M of NaOH. The low ionic strength of this media was selected to minimize nanoparticle agglomeration while still maintaining the necessary ions to support organismal growth. Experimental media was autoclaved at 120 °C for 20 min and then cooled to room temperature before use.

### 2.5. Test Organisms

*Chlamydomonas reinhardtii* was purchased from the University of Texas Culture Collection (UTEX 2243 and 2244) and cultured in TAP [50] for at least 5 days prior to use in experiments. *E. coli* was purchased from Carolina Biological Supply Company (MicroKwik culture, Burlington, NC, USA) and cultured in lysogeny broth (10 mg/L tryptone, 5 mg/L yeast extract, and 10 mg/L sodium chloride) in an incubated shaker at 37 °C for at least 24 h prior to use. *Daphnia magna* was cultured in modified International Organization for Standardization (ISO, 2012) media, consisting of calcium sulfate (120 mg/L), magnesium sulfate (120 mg/L), sodium carbonate (192 mg/L), potassium chloride (8 mg/L), and sodium selenite (0.002 mg/L). *Danio rerio* embryos were collected from a group spawn of adult wild-type zebrafish maintained at the Sinnhuber Aquatic Research Laboratory (SARL) at Oregon State University. Zebrafish embryos were developmentally staged such that each embryo was 8 h post-fertilization at the beginning of the experiment [51]. All organisms and experiments were maintained and conducted at 20.5 ± 0.5 °C with a 16:8 h light/dark photoperiod under 1690 ± 246 lux light intensity provided by full-spectrum fluorescent grow lights.

### 2.6. Experimental Design

Initial algal and bacterial cell densities in each culture were quantified using an Accuri C6 flow cytometer (BD Biosciences, San Jose, CA, USA) to allow for consistent cell densities at the beginning of the experiment. An inoculum of *C. reinhardtii* was added to 50 mL vented tissue culture flasks (Falcon 353108, Fisher Scientific, Pittsburgh, PA, USA) to achieve a starting density of 2 × 10^4^ cells/mL. *E. coli* inoculates were added to the flasks at a density of 5 × 10^5^ cells/mL. *D. magna* neonates (<24 h old) were collected from the stock culture and placed in 50% NCM for 24 h, followed by an additional 24 h in 100% NCM for acclimation prior to their use in toxicity testing. Five media-acclimated daphnids and five zebrafish embryos (8 h post-fertilization) were introduced into each experimental or control flask. Triplicate flasks were prepared for each exposure concentration (0, 0.1, or 0.5 mg Ag/L), for each of the surface chemistries (Cit, SOA-PC, SOA-PC-PT, SOA-PC-HT, and SOA-PC-HT(P)) and within each shape class (AgNSs or AgNPLs). The total volume of each flask was adjusted to 15 mL, leaving approximately 35 cc of headspace to allow for ventilation through the 0.22 µM filter cap while lying flat. Vented caps were used to equalize the air pressure inside and outside the container and minimize contamination from airborne particles and microbes. All experiments were performed in compliance with national care and use guidelines and were approved by the Institutional Animal Care and Use Committee (IACUC) at Oregon State University.

### 2.7. Toxicity Evaluations

Counts of live and dead algal and bacterial cells were conducted every 48 h by collecting 200 μL of sample from each exposure flask and adding 0.2 μL SYTOX green dead cell stain (Life Technologies, Grand Island, NY, USA) to each sample in a 96-well plate. Samples were covered and incubated in the dark for 20 min prior to being analyzed by flow cytometry. Growth rates were calculated as described previously [36]. Mortality of both *D. magna* and *D. rerio* was recorded daily; however, dead organisms were not removed from the flask or utilized for any uptake measurement at the end of the experiment. Zebrafish embryo hatching was also monitored daily. Using a modified protocol to run the experiment for 7 days allowed the hatching and development of the zebrafish embryos to be equivalent to standard embryonic zebrafish development at higher, but normally used, temperatures (i.e., 28 °C) without having to make any corrections for developmental stage [35]. At the end of the 7-day experimental period, live zebrafish embryos were removed from the experimental flasks and imaged using an Olympus SC100 high-resolution digital color camera (Olympus Corporation, Center Valley, PA, USA). Embryos were also evaluated for malformations (body axis, brain, heart, eyes, fins, jaw, trunk, and somites), physiological abnormalities (pigmentation, impaired circulation, pericardial edema, and yolk sac edema), and the ability to respond to touch. Embryos were then rinsed 3 times with ultrapure water and immediately frozen at −20 °C until digestion and silver analysis by ICP-MS could be performed.

### 2.8. ICP-MS Determination of Ag^+^ Dissolution and Organismal Uptake

Zebrafish embryos were digested in ~200 µL of ultrapure H_2_O with 200 µL of concentrated HNO_3_ (trace metal grade, Fisher Scientific) each in culture tubes (Sarstedt 55.516 series). The tubes were loosely capped and digested for 90 min at 90 °C using a heating block. After 24 h 1% HNO_3_ (trace metal grade, Fisher Scientific Pittsburg, PA, USA) was added to each tube for a total volume of 1 mL per sample. Then 5 µL of concentrated HCl (trace metal grade, Fisher Scientific, Pittsburg, PA, USA) was added to each sample to stabilize Ag in solution at 0.05%. Post-digestion, samples were transferred to 15 mL metal-free polypropylene tubes (VWR 89049-170 series) and measured undiluted. Live *Daphnia* were collected from each container and digested in ~500 µL of ultrapure H_2_O with 500 µL of concentrated HNO_3_ (trace metal grade, Fisher) each in the polypropylene culture tubes. The tubes were loosely capped, digested, and treated in the same fashion as the zebrafish embryos. *Daphnia* samples were measured following 2 times dilution (300 µL sample + 300 µL 1% HNO_3_/0.5% HCl). Algae and bacteria were digested as 1 mL bulk samples from the microcosms which were transferred to 15 mL metal-free polypropylene tubes (VWR 89049-170 series) and digested in a similar fashion to the zebrafish and daphnia. Algae and bacteria samples were measured undiluted. Dissolution samples were diluted 2 times (300 µL sample + 300 µL 1% HNO_3_/0.5% HCl) and analyzed directly. Background controls of concentrated HNO_3_ were digested by the same method as the organism samples to correct for background from digestion tubes. Silver content recovery was verified using 2 µL of a multi-element standard CEM 2, (VHG-SM70B-100) digested with concentrated HNO_3_ using the same method as the organism samples. Accuracy control was maintained with the use of a NIST SRM 1683f prepared as 2 times dilution (1 mL of 1% HNO_3_/0.5% HCl + 1 mL of NIST SRM 1683f) to ensure the accuracy of the standard calibration curve.

Inductively coupled plasma mass spectroscopy (ICP-MS) analysis was performed using an Agilent 7700x equipped with an ASX 500 auto sampler. The system was operated at a radio frequency power of 1550 W, an argon plasma gas flow rate of 15 L/min, and an Ar carrier gas flow rate of 0.9 L/min. Silver was measured in NoGas mode. Data were quantified using a 9-point (0, 0.5, 1, 2, 5, 10, 20, 50, 100 ppb (µg/kg)) calibration curve using a single-element standard (Ag, (VHG-LAGN-100, Lot# 404-0117-1)) in 1% HNO_3_/0.5% HCl. For each sample, data were acquired in triplicates and averaged. A coefficient of variance (CoV) was determined from frequent measurements of a sample containing ~10 ppb Ag. An internal standard (Sc, Ge, Bi) continuously introduced with the sample was used to correct for detector fluctuations and to monitor plasma stability.

### 2.9. Statistical Analyses

Statistical analyses were performed using Sigma Plot version 13.0. Fisher’s exact test was used to compare specific developmental endpoints between treatment and controls in the embryonic zebrafish assay. Analysis of variance (ANOVA) was used to evaluate differences among treatment groups across equivalent concentrations. Differences were considered statistically significant at *p* ≤ 0.05 for all analyses.

## 3. Results and Discussion

### 3.1. AgNP Synthesis and Characterization

Our previous studies of spherical and triangular-plate AgNPs before and after coating with Cit ligands or SOA-PC and SOA-PC-Thiol (PT or HT) membranes show that the robustness of the nanoparticles towards surface oxidation and Ag^+^ ion release is dependent on the complexity of the ligand architecture shielding the AgNP surface [38,39]. In a previous study, we showed the total Ag^+^ ion dissolution in simple fishwater over a one-week period to be low and that the particle stability was ranked from least to most robust in the following order: Cit < SOA-PC < SOA-PC-PT < SOA-PC-HT ≈ SOA-PC-HT (P) [39]. However, the hybrid lipid-coated Ag-SOA-HT were stable for more than 4 weeks. TEM analysis of the particles used in this study shows the primary core size of the most stable hybrid lipid-coated AgNSs was 13.6 ± 4.4 nm with a localized surface plasmon resonance (LSPR) band of λ_max_ 426 nm and the AgNPLs had an average side length of 30.2 ± 6.3 nm with LSPR bands at λ_max_ 440 nm and 650 nm (Figure 1A,B and Figure S1), which is consistent and within the standard deviation of batches published previously [38].

**Figure 2:**
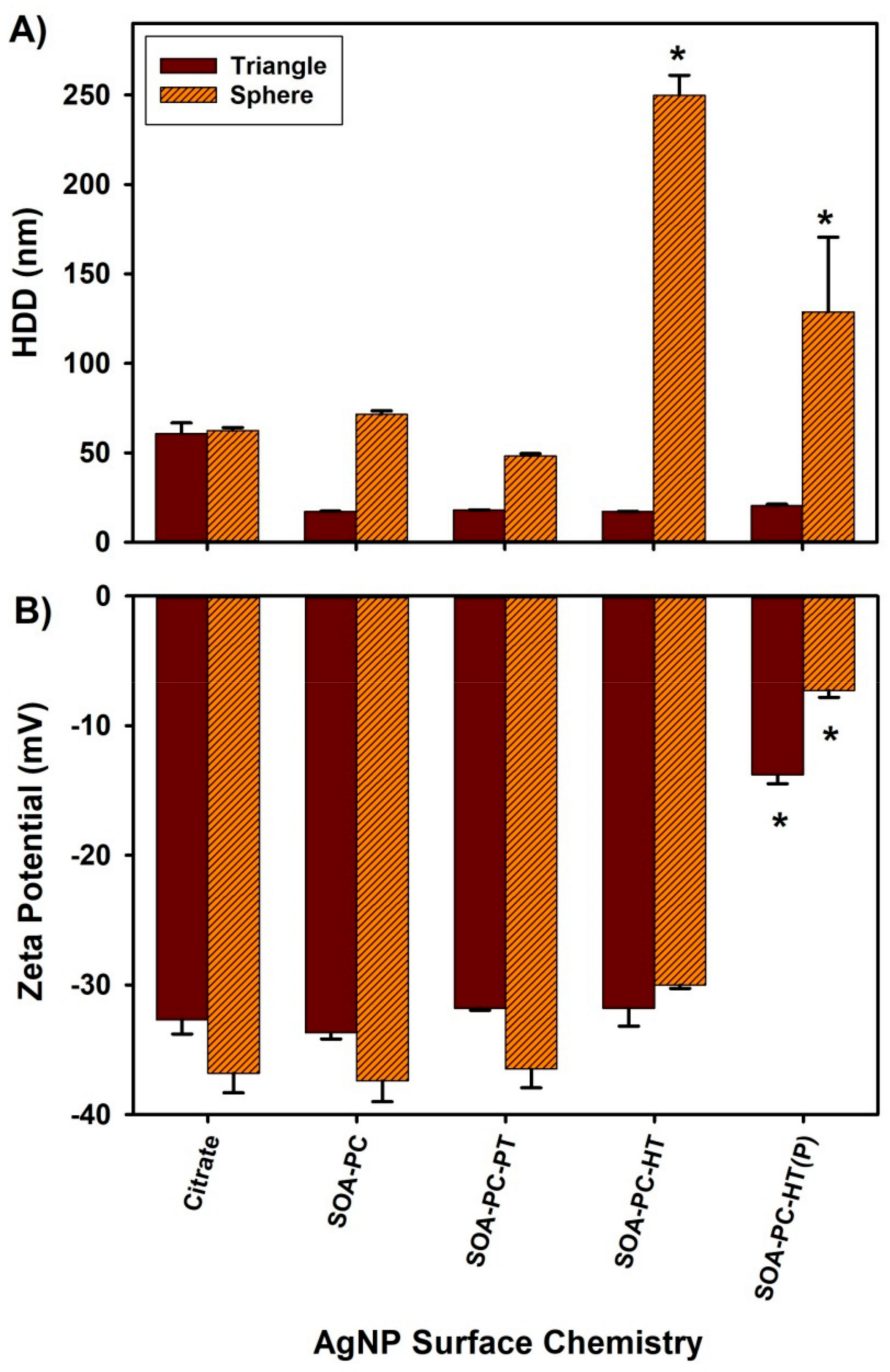
Dynamic light scattering results for engineered AgNPs suspended in NCM at 0.5 mg Ag/L. (**A**) Hydrodynamic diameter (HDD) and (**B**) zeta potential. Asterisk (*) indicates a significant difference from citrate-capped nanoparticles (*p* ≤ 0.05). Error bars represent the standard error between replicate measurements (n = 3).

We also evaluated each type of nanomaterial shape with corresponding surface chemistries to determine the effect of the NCM media components on AgNP agglomeration by monitoring changes in hydrodynamic diameter (HDD) and ZP by DLS. Cit-capped AgNPLs show an average HDD of 61 ± 6 nm, larger than the primary core nanoparticle size of 30 nm determined by TEM suggesting some possible nanoparticle-nanoparticle interactions, while the other surface chemistries HDD ranged from 17 ± 1 to 20 ± 1 nm (Figure 2A). Thus, most of the HDDs of the AgNPLs are lower than the primary particle size determined by TEM, likely due to the non-spherical nature of these particles interacting with the DLS laser beam and the potential for some smaller AgNSs to also be present following the synthesis. AgNSs showed minimal agglomeration with Cit or when capped with SOA-PC or PC-SOA-PT membranes with average HDD ranging from 48 ± 1 to 62 ± 2 nm. In contrast, the AgNPLs with PC-SOA-HT showed significant agglomeration (129 ± 42 to 250 ± 11 nm average HDD) in NCM (Figure 2A).

ZPs for both shape types were negative with similar values among the various surface chemistries (Figure 2B). All nanoparticles except the AgNPs with PC-SOA-HT (P) showed ZPs in the range of −30 to −37 mV indicating moderate stability, while the purified AgNPs with PC-SOA-HT membrane in both shape profiles (AgNSs and AgNPLs) had significantly decreased zeta potential (−7 and −13 mV, respectively) compared to the other nanoparticles. The reduction in the ZP for the purified SOA-PC-HT nanoparticles is due to the removal of free ligands not bound to the surface allowing for increased interaction of the particles with the NCM. Note that DLS is driven by Derjaguin, Landau, Verwey, and Overbeek (DLVO) theory, thus the results are based on the assumption that the AgNPs are spherical [52]. Given that our AgNPLs are not spherical, one must interpret DLS results with caution, especially when making statistical comparisons between spherical and triangular-plate-shaped AgNPs. Furthermore, in order to determine ZP accurately, it is imperative to measure the electrophoretic mobility of nanoparticles with precision. While there is a potential for misinterpretation of scattered laser light due to the movement of AgNPL under Brownian motion, it cannot be ignored. Nevertheless, the remarkable similarity in the reductions in ZP observed in both spherical and triangular SOA-PC-HT (P) nanoparticles (Figure 2B) provides compelling evidence that the measures do indeed represent the trends of the nanoparticle surface charge and electrophoretic mobility relative to the experimental media. The results from the measures of HDD and ZP by DLS show that regardless of shape, the Cit, SOA-PC, and SOA-PC-PT modified surfaces are all stable suspensions in the experimental NCM. In contrast to the AgNPLs, there are considerably more nanoparticle-nanoparticle interactions that result in agglomeration with the purified and unpurified AgNSs with SOA-PC-HT surface chemistry (Figure 2). This agglomeration of the unpurified and purified AgNSs with SOA-PC-HT suggests there are interactions between the surface of the AgNSs with SOA-PC-HT and the NCM, which was not observed when these same nanoparticles were placed in simpler salt and fishwater media solutions [40]. Interestingly, the only significant changes in ZP are observed with the AgNSs and AgNPLs with SOA-PC-HT (P), yet no agglomeration of the AgNPLs occurred. This suggests that the change in ZP for the spherical AgNPs with SOA-PC-HT (P) may be related to an increased ability of the purified sample to interact with the ions in the NCM, compressing the double layer surrounding the nanoparticles and thus reducing the ZP while still being more stable in suspension than the much smaller AgNPs. We estimate that if all 0.5 mg of Ag is converted to AgNSs (13.6 nm) or AgNPLs (30 nm), then based on their diameter from TEM and packing of Ag atoms into a sphere or plate, there would theoretically be more spheres in the sample than the AgNPLs. This would explain the greater nanoparticle-nanoparticle interactions seen in NCM with the AgNSs (9.4 × 10^12^ nanoparticles) versus the AgNPLs (3.2 × 10^11^ nanoparticles). However, this is a rough approximation since the AgNPL sample comprises a mixture of spheres (35%), rods (1%), and triangles (64%) based on previous TEM analysis [38].

### 3.2. AgNP Surface Oxidation and Dissolution Studies

**Figure 3:**
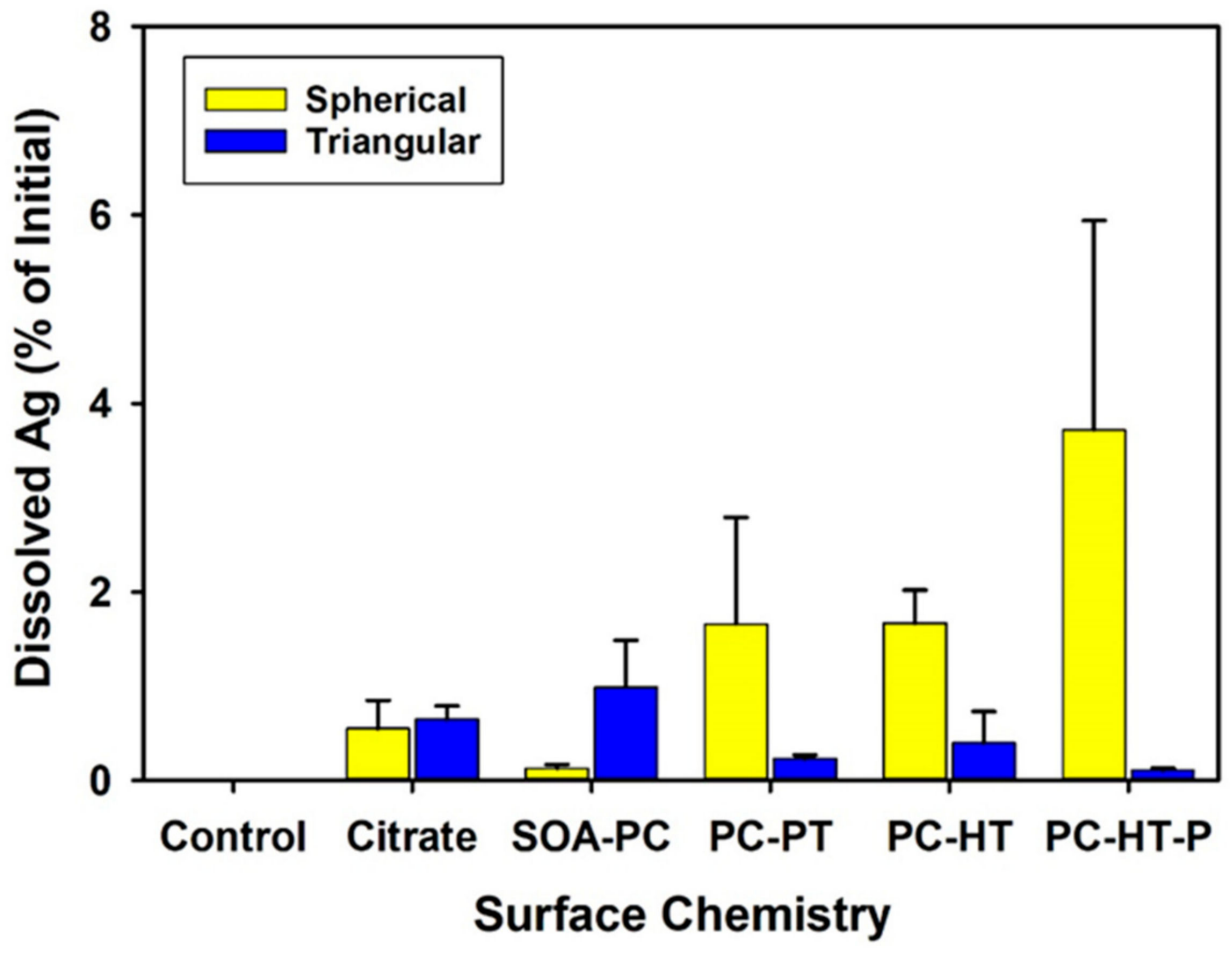
The amount of dissolved Ag^+^ ion found in the microcosm media after 7 days with organisms present in the flasks. None of the measured amounts are significantly different than the control flasks that did not contain any AgNPs and only had the test organisms (*p* ≤ 0.05). Error bars represent the standard error between replicate flasks (n = 3 per treatment).

The dissolution of the AgNSs and AgNPLs with varying surface chemistries was measured in the NCM by ICP-MS to correlate the impact of the dissolved salts and light cycles on AgNP dissolution without the organism’s presence (abiotic dissolution) and at the end of the experiment to quantify the amount of freely dissolved Ag^+^ ion in the presence of the organism’s (biotic dissolution). After 5 days, the amount of abiotic Ag^+^ ion dissolution was less than 4% of the 0.5 mg Ag/L initially placed into the media on day 1 (Figure S2) for all AgNPs with no significant differences from control flasks without AgNPs present. As such, there were no differences in abiotic dissolution in the NCM regardless of shape or surface chemistry. Dissolution rates were similarly low across all surface chemistries in the biotic exposures for both AgNP shapes and were also not significantly different from control exposures without AgNPs (Figure 3). This suggests that the surface modifications block oxidative dissolution and the release of Ag^+^ ions over the 7-day experiment. It is not likely that dissolved silver was precipitated as other forms of particulate silver, as Visual-MINTEQ analysis from previous work with AgNPs using the same experimental NCM shows that concentrations were far below the threshold for precipitation of other species [37].

### 3.3. Impacts on Bacterial Growth

**Figure 4:**
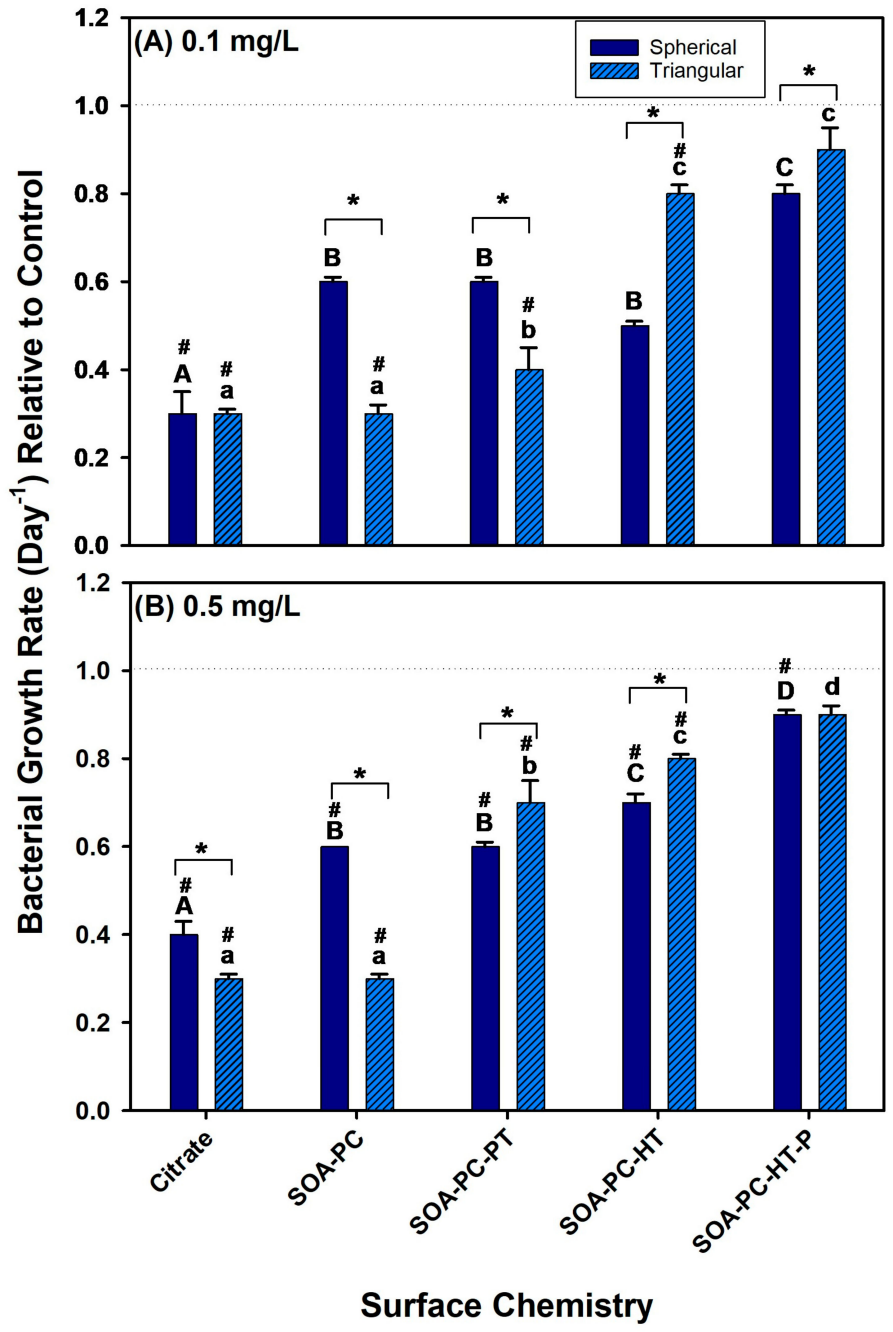
The bacterial growth rate for various surface-modified AgNSs and AgNPLs over the 7-day experimental period at (**A**) 0.1 mg Ag/L and (**B**) 0.5 mg Ag/L. Asterisks (*) represent significant differences between shapes at a given concentration, letters represent significant differences between surface chemistries with each shape, and hash (#) represents significant differences from control flask growth rates. Error bars represent the standard error between replicate flasks (n = 3 per treatment).

To evaluate the effect of the AgNPs with various coatings on bacterial growth, their growth was monitored during exposure to 0.1 and 0.5 mg Ag/L over the 7-day experimental period. At low-concentration exposures (0.1 mg Ag/L) the growth rate of bacteria was reduced relative to control in all the AgNPLs exposures except the SOA-PC-HT (P), which was not significantly different from the control (Figure 4A). The low-concentration AgNSs also showed similar trends in the reduction of bacterial growth rates; however, only the Cit-AgNPs were significantly lower than control bacterial growth rates. This suggests that Cit-AgNPs have the most impact on the growth rate of the bacteria. Furthermore, the growth rate of bacteria was reduced relative to control for all of the high concentration (0.5 mg Ag/L) exposures, regardless of nanoparticle shape or surface chemistry except for the purified AgNPLs with SOA-PC-HT (Figure 4B). The fact that the purified AgNPLs were the only material to not significantly decrease bacterial growth rates relative to control suggests the additional purification may have played a role, and part of the decreased growth rates observed in this study was due to the residual reactant impacts on bacterial growth, rather than the release of silver ions. Overall, the bacterial growth rates in the presence of the AgNPs were lower than control bacterial growth rates suggesting some antimicrobial activity of the AgNPs even at low Ag^+^ ion dissolution rates. The decreased growth rate was more significant with the Cit-capped AgNPs and then decreased as the complexity of the ligand architecture increased to shield the AgNPs from surface oxidation and Ag^+^ ion release (Figure 4). Thus, given the low Ag^+^ ion dissolution and largest negative impacts on bacterial growth rate in the simpler surface chemistries such as Cit, the coatings with the SOA, PC, and hydrophobic thiols may also prevent the bacteria from interacting with the AgNPs in a way that leads to bacterial cell death. From this study, the surface chemistry, more so than shape, is a determinant of impacts on bacterial growth rates. Despite this fact, one must use caution when describing this trend as the bacterial live cell counts fluctuated daily (Figures S3 and S4) and may reflect the impacts in other organisms (i.e., *Daphnia* death); thus, it is possible that, given a longer experimental period, a more stabilized growth rate may have been achieved that may differ from the results presented here.

### 3.4. Impact on Algal Growth Rates

**Figure 5:**
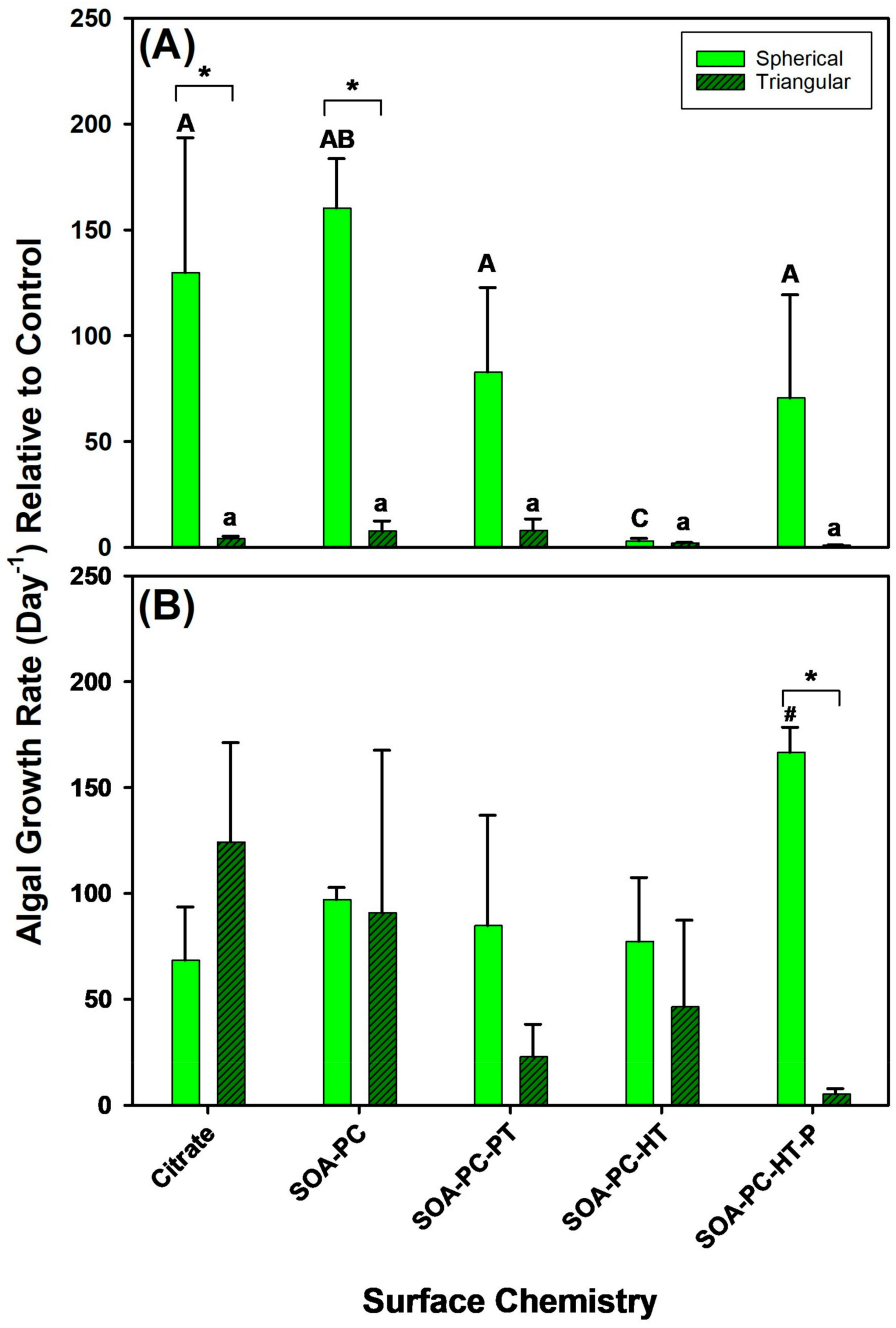
The algal growth rate for various surface-modified AgNSs and AgNPLs over the 7-day experimental period at (**A**) 0.1 mg Ag/L and at (**B**) 0.5 mg Ag/L. Asterisks (*) represent significant differences between shapes at a given concentration, letters represent significant differences between surface chemistries with each shape, and hash (#) represents a significant difference from control flask growth rates. Error bars represent the standard error between replicate flasks (n = 3 per treatment).

To understand the impact of AgNP surface chemistry and shape on algal growth rates, their growth was also monitored upon exposure to 0.1 mg Ag/L and 0.5 mg Ag/L over the 7-day experimental period (Figure 5). At the lowest Ag concentration (0.1 mg Ag/L), significant changes in the growth rate of AgNPLs with Cit and SOA-PC are observed relative to spherical Ag-SOA-PC, while the other surface chemistries had similar growth rates between nanoparticle shapes (Figure 5A). Within shapes, there were no significant differences in growth rate among AgNPLs; however, Ag-SOA-PC had increased growth rates and unpurified Ag-SOA-PC-HT had significantly decreased growth rates. At high concentration exposures (0.5 mg Ag/L) the only difference in algal growth rate is observed with the purified spherical Ag-SOA-PC-HT, which showed a significantly increased growth rate relative to control for AgNSs with other surface chemistries. The algal growth rate was significantly slowed with the triangular-shaped purified Ag-SOA-PC-HT (Figure 5B and Figures S5 and S6). In contrast to the bacterial growth, the algal growth rates are similar to control growth rates except for the AgNSs with SOA-PC-HT (P) coating (Figure 5). The algal growth rates were highly variable within replicate flasks, leading to large error terms and a lack of significant differences from control algal growth rates, even though an increase in the number of live algal cells was frequently observed (Figures S5 and S6). This increase in live algal cells did not correlate with daphnia survival, suggesting that this increase may be related to nutrient availability and the cyclical nature of the bacterial population over time (Figures S3 and S4).

### 3.5. Algal and Bacterial Silver Uptake

**Figure 6:**
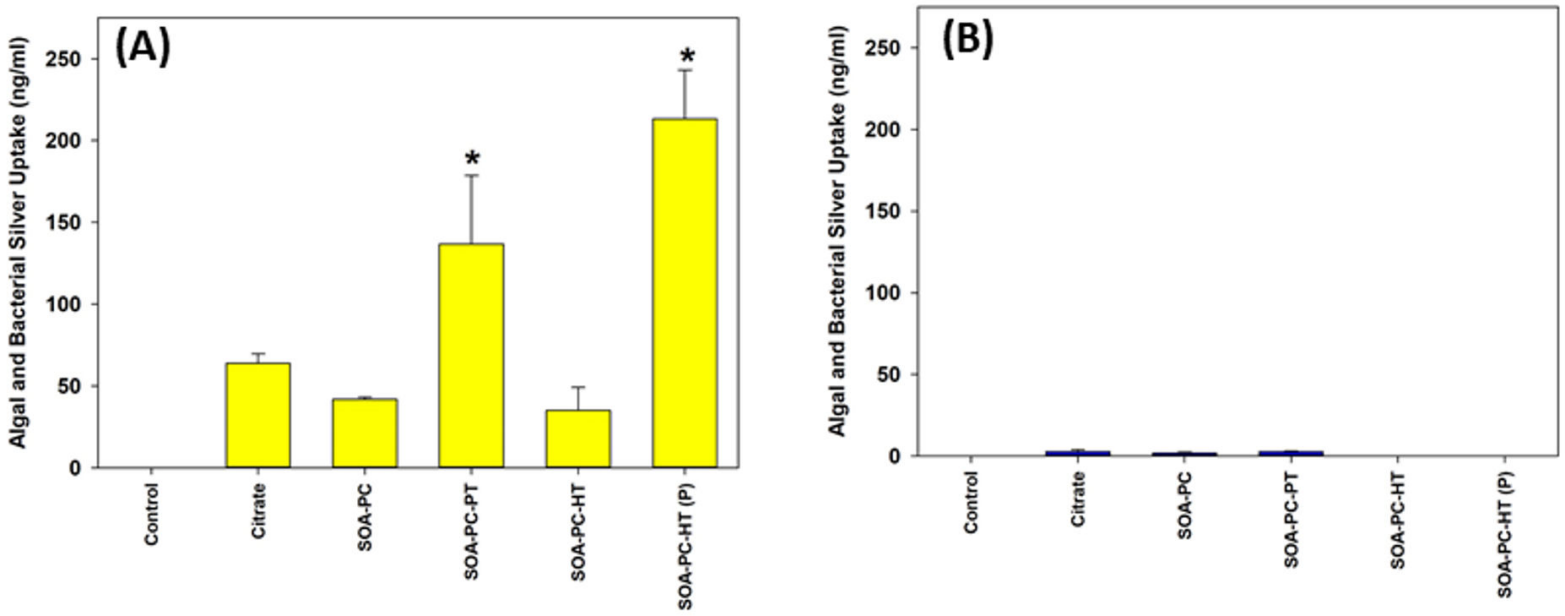
Uptake of silver by algal and bacterial cells (combined) as determined by ICP-MS following exposure to (**A**) AgNPLs and (**B**) AgNSs over the 7-day experimental period at 0.5 mg Ag/L for all surface chemistry variations and control flasks (with no AgNPs present). Asterisks (*) represent significant differences between control and AgNP-treated exposures (*p* < 0.05). Error bars represent the standard error between replicate flasks (n = 3 per treatment).

To evaluate the effect of shape and surface chemistry on silver uptake, the quantification of total Ag was performed by ICP-MS after digesting combined samples of algae and bacteria from the microcosms. The uptake of Ag by algal and bacterial cells was measured after 7 days of exposure to both AgNSs and AgNPLs with all the surface chemistries. Ag uptake is dramatically higher for AgNSs in comparison to AgNPLs, regardless of surface chemistry (Figure 6 and Figure S7). However, a significant increase is observed with spherical Ag-SOA-PC-HT (P) and Ag-SOA-PC-PT nanoparticles. Since the uptake of Ag by the bacteria and algae was measured as a combined sample of everything suspended in the water column except for *Daphnia* and zebrafish embryos, this sample represents not only the grown algae and bacteria but also those microbes associated with the 7-day experiment. Even though the exposure to AgNSs at 0.5 mg Ag/L is the only exposure to have accumulation significantly higher than control, the values measured are approximately 10 times higher than in the 0.1 mg Ag/L exposure, suggesting a high affinity of either algae or bacteria for the AgNSs with a lipid-coating. This cellular affinity for the AgNPs is drastically reduced in the AgNPL exposures, suggesting that shape is a determinant in the amount of silver taken up by algae and/or bacteria.

### 3.6. Daphnia Toxicity and Silver Uptake

**Figure 7:**
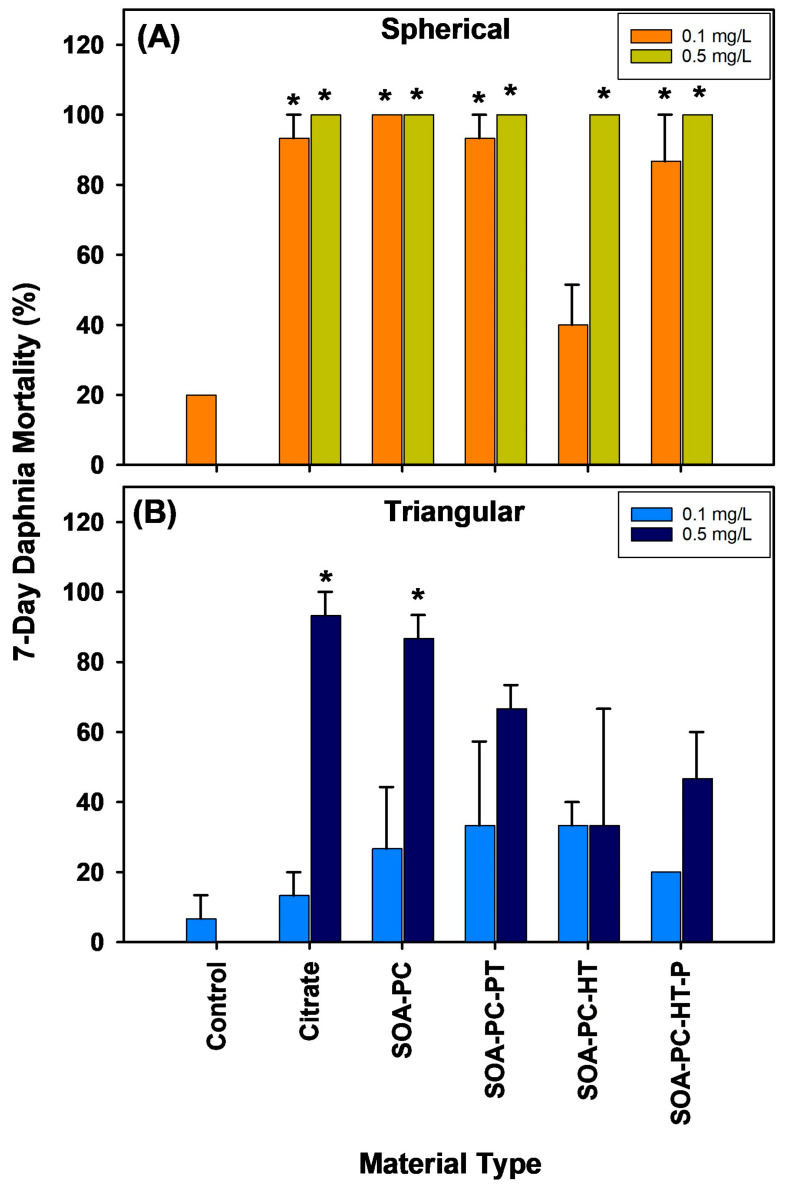
Percent mortality of daphnia exposed to AgNPs with varying shapes and surface chemistries over the 7-day experimental period. Panel (**A**) shows the spherical particle mortality, while panel (**B**) shows the triangular particle daphnia mortality. Asterisks (*) indicate a significant difference from the control mortality rate without nanoparticles present. Error bars represent the standard error between replicate flasks (n = 3 per treatment).

*Daphnia* survival in the flasks exposed to each shape of AgNPs across all the various surface chemistries was recorded daily (Figures S8–S11). The overall mortality rate for daphnia over the 7-day experiment varied dramatically depending on the shape of AgNPs with AgNSs showing significantly higher *Daphnia* mortality than is found for AgNPLs with the same surface chemistry and mass of Ag (Figure 7). Both concentrations of AgNSs (0.1 and 0.5 mg Ag/L) caused significant *Daphnia* mortality over 7 days for all surface chemistries except the low concentration of SOA-PC-HT (Figure 7A). In contrast, the AgNPLs only caused significant mortality in the highest concentration exposures of Cit and SOA-PC (Figure 7B).

At the end of the 7-day exposure, surviving daphnids were rinsed 3 times then digested in HNO_3_ and analyzed for Ag uptake by ICP-MS (Figure S12). Note that limited data are available for the AgNSs due to the increased *Daphnia* mortality. However, AgNPLs with either Cit or SOA-PC-HT showed the highest uptake. *Daphnia* mortality being overall higher in the flasks containing AgNSs, regardless of surface chemistry, may be related to the similarity between the spherical AgNPs and the food typically ingested by daphnids. Although the high concentration AgNPLs with the simpler surface chemistries (Cit or SOA-PC) also resulted in high *Daphnia* mortality, this is not observed for the more complex surface chemistries with the thiols (SOA-PC-thiol), thus the impacts are possibly related to the feeding behavior of the daphnids, favoring the AgNSs relative to AgNPLs. This is supported by the increased *Daphnia* mortality in virtually all of the flasks with AgNSs. This is evident even in the low-concentration exposures to AgNSs, which resulted in nearly 100% mortality of *Daphnia*, while the same concentration of the AgNPLs did not result in any significant *Daphnia* mortality. The shape-dependent toxicity of AgNPs, with AgNSs having the highest impact, has also been shown for plastic microparticles, which supports our findings [53,54]. The high mortality of the *Daphnia* in the AgNS exposures precluded thorough analysis of the Ag uptake by those daphnids (Figure S12); however, the higher uptake by surviving daphnids in the AgNPL exposures could support the ability of *Daphnia* to conduct shape-dependent ingestion of nanoparticles. It may be that AgNPLs, although filtered from the water column, are not ingested and instead remain in the filter setae of the daphnids and are not moved to the mouth for ingestion, resulting in the higher accumulation of Ag. In contrast, AgNSs may be ingested and digested to a greater extent than AgNPLs resulting in higher toxicity. It is also possible, however, that the AgNSs being slightly smaller than the AgNPLs can be internalized from the digestive tract into the daphnia simply by way of their smaller size. This should be the subject of future investigations into the shape-dependent uptake and particulate feeding behavior of daphnids.

When considering the survival of the daphnia, it is important to also consider the changes in algal growth rates that were found to be related to AgNP shape in the low-concentration exposures which differed from the higher-concentration exposures where shape did not have a significant impact on algal growth rates. This is interesting as *Daphnia* mortality is highest upon exposure to AgNSs and significant at both concentrations (Figure 7). Given that algal growth rates are slower than bacterial growth rates and that surviving daphnids would be feeding on the algae, it is expected that AgNS exposures will have the highest algal growth rates due to the lack of grazing. However, a significant increase in algal growth is not observed relative to control flasks. A similar finding is also seen at the high concentration level of AgNPLs with Cit or SOA-PC coatings resulting in nearly 100% mortality of the *Daphnia*, yet no significant increase in algal growth rates was found in those flasks. Only the high concentration exposure of AgNSs with SOA-PC-HT (P) coating significantly increased algal growth relative to control flasks, so algal growth rate did not seem to be related to *Daphnia* survival and may have been more related to nutrient availability or some other limiting factor.

### 3.7. Zebrafish Toxicity and Silver Uptake

**Figure 8:**
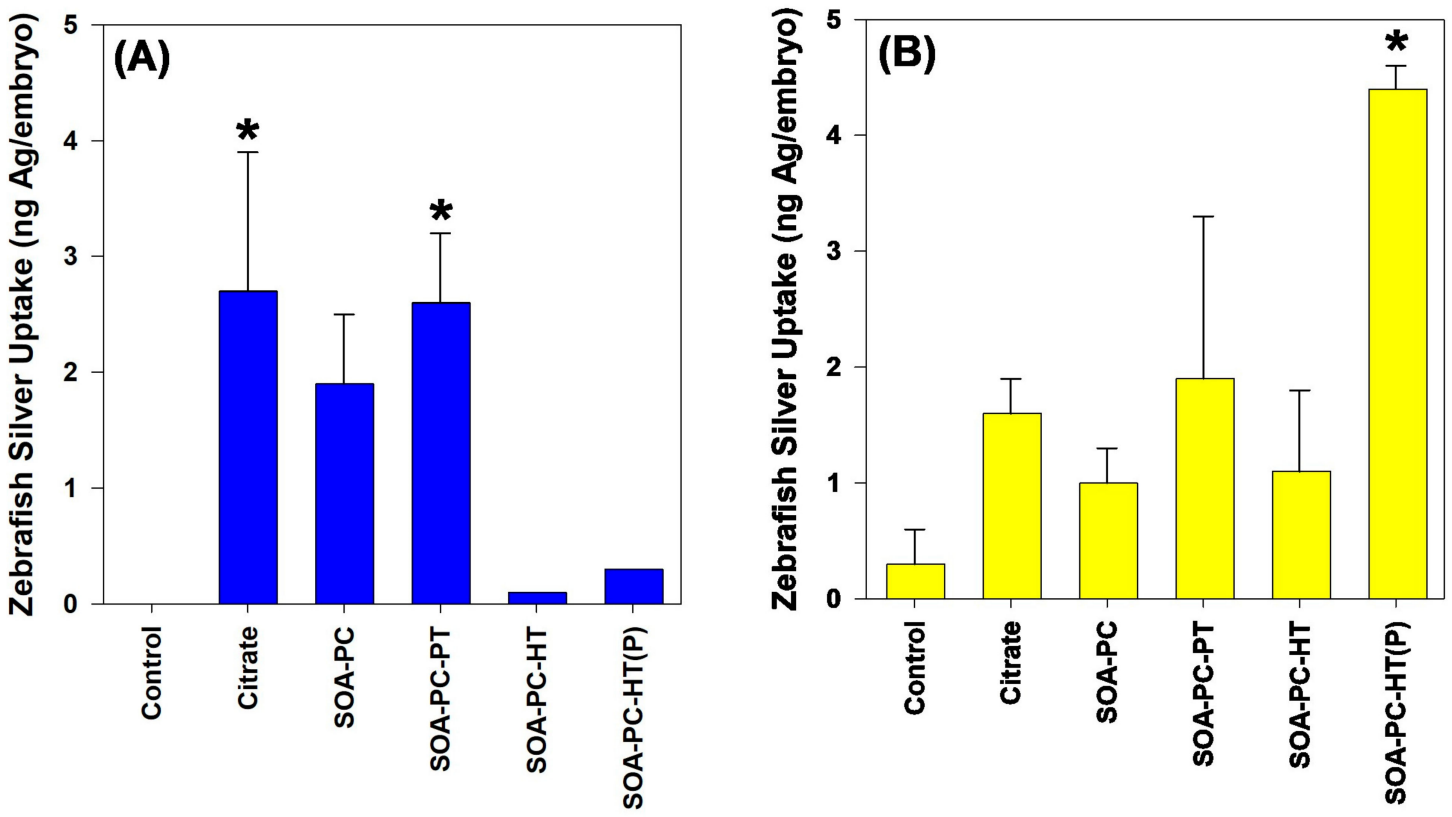
Silver uptake by zebrafish embryos as determined by ICP-MS after 7 days of exposure to 0.5 mg Ag/L. Panel (**A**) represents the AgNPLs and panel (**B**) shows silver uptake following exposure to AgNSs. Asterisk (*) indicates significance relative to control embryo silver content. Error bars represent the standard error between replicate flasks (n = 3 per treatment).

The mortality rate for zebrafish exposed to both shapes of AgNPs was not significantly different than the control mortality rate over the 7-day exposure. No impact on the hatching rate is noticed for any shapes or surface chemistries of AgNPs (Figure S13). In addition, no other morphological or developmental abnormalities were noticed at the end of the exposure. Following the exposure, surviving zebrafish embryos from the 0.5 mg Ag/L exposures were washed 3 times with ultrapure H_2_O and digested in trace-metal grade HNO_3_ for the determination of total Ag content by ICP-MS. Only AgNPLs with Cit and SOA-PC-PT coatings resulted in any significant accumulation of Ag relative to control embryos (Figure 8). In contrast, only the purified AgNSs with HT resulted in any significant uptake of silver during the 7-day exposure.

The low mortality of zebrafish embryos even at the highest concentration of Ag (0.5 mg Ag/L) is not surprising, given the low dissolution rates of the nanoparticles and the fact that embryos are not actively feeding during the duration of these experiments. In addition, other studies in zebrafish have found that exposure to Cit-capped AgNPs of a similar size to the AgNSs does not cause mortality in zebrafish embryos until concentrations exceed those used in these experiments [55,56]. In addition, our previous work found that the concentrations used in these experiments are not likely to cause toxicity to zebrafish embryos [40]. Interestingly, despite no mortality occurring, there are differences in the uptake of AgNPs by the zebrafish embryos during their development. AgNPLs consisting of Cit and SOA-PC-PT showed significant uptake relative to control embryos (Figure 8), which correlates with the non-significant but elevated Ag uptake for the algae and bacteria from those flasks (Figure 6). Overall, the uptake of Ag by the zebrafish follows the same trends as was measured in the algae and bacterial Ag uptake, suggesting that much of the Ag is taken up by the embryos and this could be related to bacterial films on the embryo tissues following hatching.

## 4. Conclusions

Given the widespread use of AgNPs and Ag^+^ in various commercial products, it is essential to assess their potential impact on the environment and human health. Therefore, a comprehensive risk assessment of these materials is of utmost importance. Evaluating these materials poses a challenge since the dissolution of Ag^+^ ions is a confounding variable in toxicity. As a result, it has been difficult to understand the risk of the AgNP itself, and how its physicochemical characteristics affect toxicity. Additionally, most studies have been conducted on single organisms and not in complex environments with multiple species where whole communities can be affected by material behavior. Given this need, we assessed how the physicochemical properties of AgNPs with five different surface chemistries affect their uptake and toxicity in bacteria, algae, *Daphnia*, and embryonic zebrafish. The surface chemistry of AgNPs varied from the least complex to increasing complexity to protect the AgNP from surface oxidation and Ag^+^ ion dissolution.

The results of this study confirm that the lipid-coated AgNPs did not release any significant amounts of Ag^+^ ions, but they exhibited the greatest toxicity to *D. magna*, which was the most sensitive species in the microcosm. The findings of this study emphasize the influence of particle shape and surface chemistry in determining both the level of exposure and the mechanism of toxicity. Moreover, it shows how the impact on the most sensitive species can directly affect other organisms in the ecosystem. It is worth noting that surface chemistry seems to play the largest role in determining the ecotoxicity of AgNPs relative to the particle size or shape; however, all of these factors interact with differential proportions in different organisms.

The shape of nanomaterials may alter NP fate and transport in the ecosystem or inside organisms, with the potential of some shapes accumulating where digestive or anoxic environments could influence their stability and dissolution relative to particles with different shapes. Surface functionalization can stabilize NPs and prevent dissolution as was seen in this study but can also impact the outermost charge on the particle, making it more or less likely to interact with oppositely charged membranes in the ecosystem. Overall, the study demonstrates that we can manipulate the shape and surface chemistry of AgNPs to achieve specific objectives necessary to further understand and mitigate the risks associated with silver nanoparticles.

## Data Availability

Data are contained within the article and Supplementary Materials.

## Supplementary Materials

The following supporting information can be downloaded at: https://www.mdpi.com/article/10.3390/nano14080654/s1, Figure S1: Representative TEM distribution and histogram analysis of hybrid lipid-coated AgNPLs. TEM scale bar is 50 nm. Figure S2: Amount of dissolved silver found in solution after 7 days in abiotic particle exposure media as determined by ICP-MS; Figure S3: Bacterial growth represented as the change in the number of live cells over the 7-day experiment for spherical AgNPs with varying surface chemistries at 0.1 mg Ag/L and at 0.5 mg Ag/L; Figure S4: Bacterial growth represented as the change in the number of live cells over the 7-day experiment for triangular AgNPs with varying surface chemistries at 0.1 mg Ag/L and at 0.5 mg Ag/L; Figure S5: Change in the number of live algal cells following exposure to spherical AgNPs at 0.1 mg Ag/L and 0.5 mg Ag/L over the 7-day experimental period; Figure S6: Change in the number of live algal cells following exposure to triangular AgNPs at 0.1 mg Ag/L and 0.5 mg Ag/L over the 7-day experimental period; Figure S7: Silver uptake in algal and bacterial cells following 7-day exposure to 0.1 mg Ag/L of lipid coated triangular and spherical nanoparticles with varying surface chemistries; Figure S8: Daily mortality rate (%) for daphnids exposed to AgNSs at 0.1 mg Ag/L over the 7-day experimental period; Figure S9: Daily mortality rate (%) for daphnids exposed to AgNPLs at 0.1 mg Ag/L over the 7-day experimental period; Figure S10: Daily mortality rate (%) for daphnids exposed to AgNSs at 0.5 mg Ag/L over the 7-day experimental period; Figure S11: Daily mortality rate (%) for daphnids exposed to AgNPLs at 0.5 mg Ag/L over the 7-day experimental period; Figure S12: Silver uptake by Daphnia surviving the 7-day exposure as determined by ICP-MS; Figure S13: Hatching rate for zebrafish embryos exposed to spherical or triangular AgNPs at 0.5 mg Ag/L over the 7-day experimental period.
