# Supplementary material for: Impacts of Differentially Shaped Silver Nanoparticles with Increasingly Complex Hydrophobic Thiol Surface Coatings in Small-Scale Laboratory Microcosms
Source: Nanomaterials (Basel). 2024 Apr 9;14(8):654. doi: 10.3390/nano14080654 (PMC11054431; doi:10.3390/nano14080654)
Supplement: Supplementary file 1 [file nanomaterials-14-00654-s001.zip › nanomaterials-2907613-supplementary.pdf]

Impacts of Differentially Shaped Silver Nanoparticles with Increasingly Complex Hydrophobic Thiol Surface Coatings in Small-Scale Laboratory Microcosms

Bryan J. Harper 1, Arek M. Engstrom 1,2, Stacey L. Harper 1,2,3,* and Marilyn R. Mackiewicz 4,*
1 Department of Environmental and Molecular Toxicology, Oregon State University, Corvallis, OR 97331, USA; bryan.harper@oregonstate.edu (B.J.H.); arek.engstrom@gmail.com (A.M.E.)
2 School of Chemical, Biological, and Environmental Engineering, Oregon State University, Corvallis, OR 97331, USA
3 Oregon Nanoscience and Microtechnologies Institute, Corvallis, OR 97331, USA
4 Department of Chemistry, Oregon State University, Corvallis, OR 97331, USA
* Correspondence: stacey.harper@oregonstate.edu (S.L.H.); marilyn.mackiewicz@oregonstate.edu (M.R.M.)

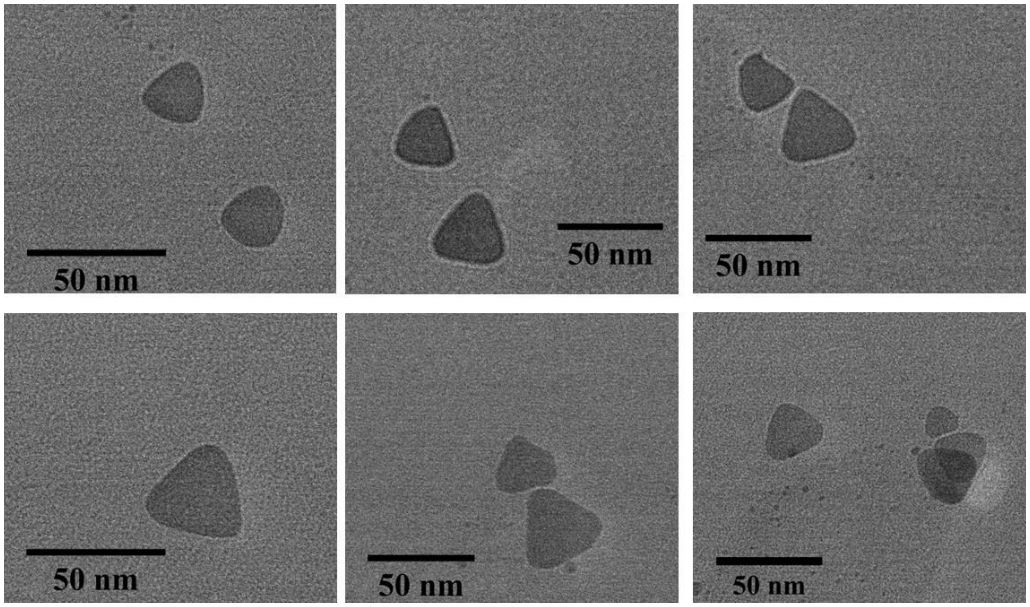

Figure S1. Representative TEM distribution and histogram analysis of hybrid lipid-coated AgNPLs. TEM scale bar is 50 nm.

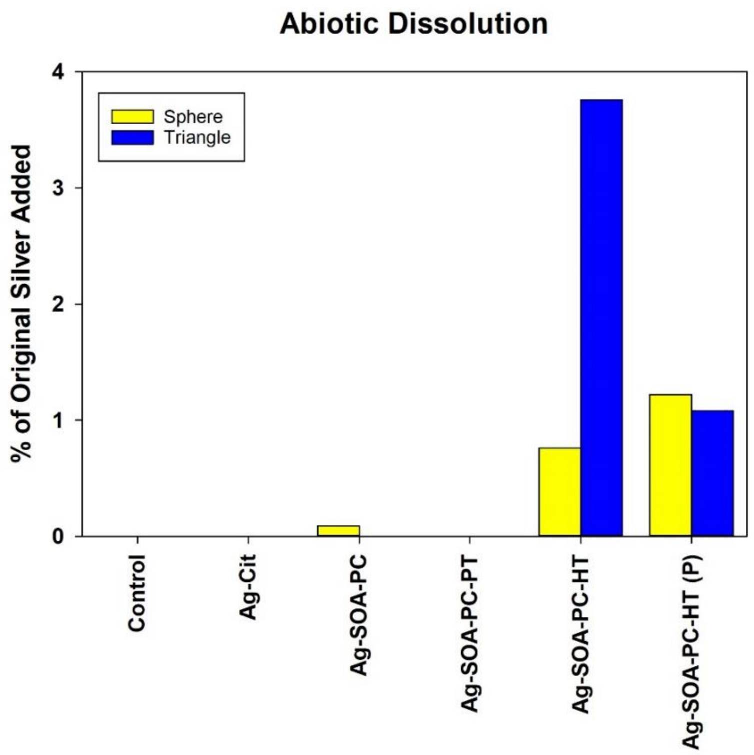

Figure S2: Percentage of original 0.5 mg Ag/L added to each flask found in the microcosm media as dissolved Ag^{+} after 7 days without organisms present in the flasks. None of the measured amounts are significantly different than the control (no AgNPs present).

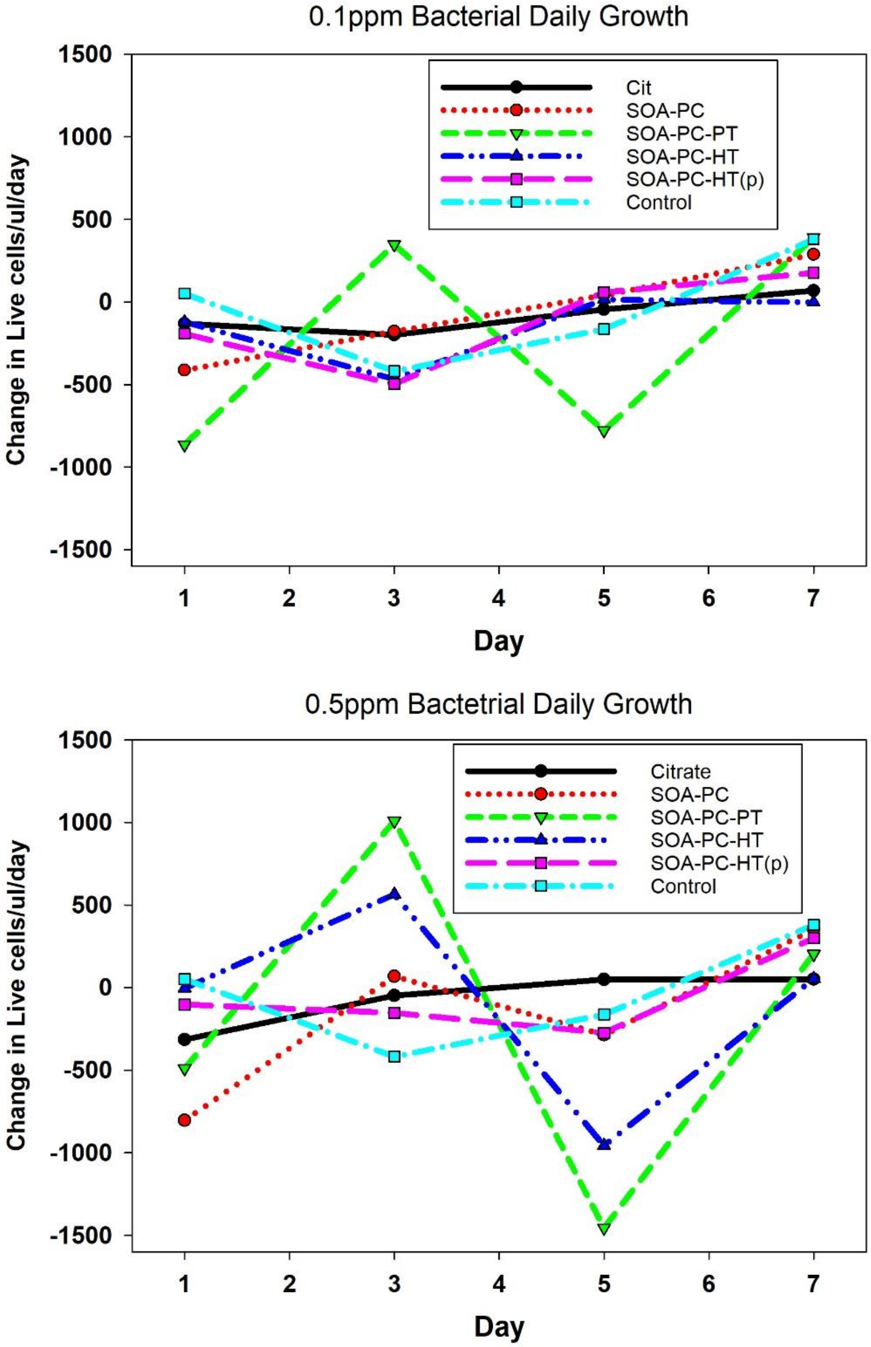

Figure S3: Bacterial growth represented as the change in the number of live cells over the 7-day experiment for spherical AgNPs with varying surface chemistries at 0.1 and at 0.5 mg Ag/L

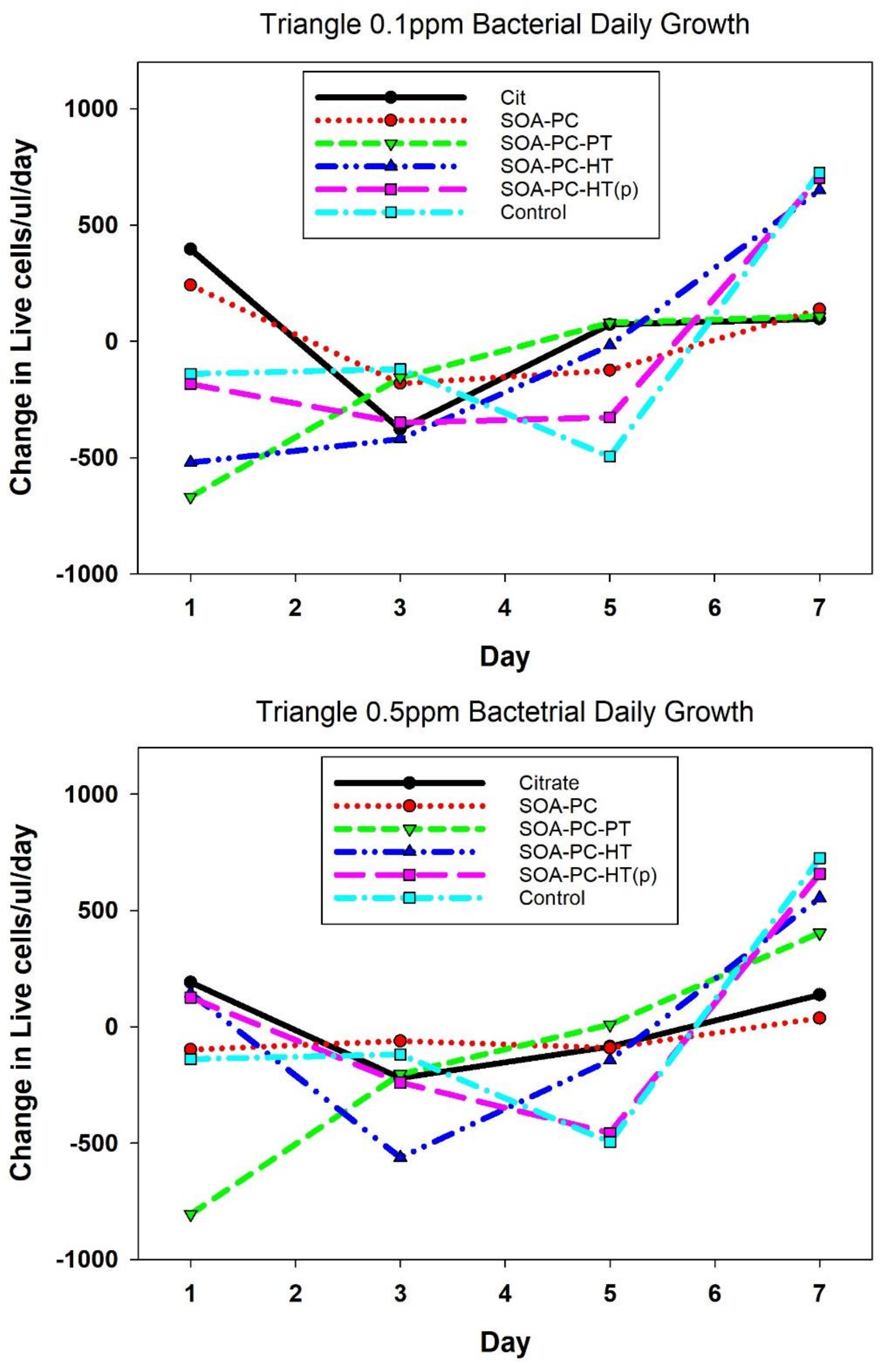

Figure S4: Bacterial growth represented as the change in the number of live cells over the 7-day experiment for triangular AgNPs with varying surface chemistries at 0.1 and at 0.5 mg Ag/L.

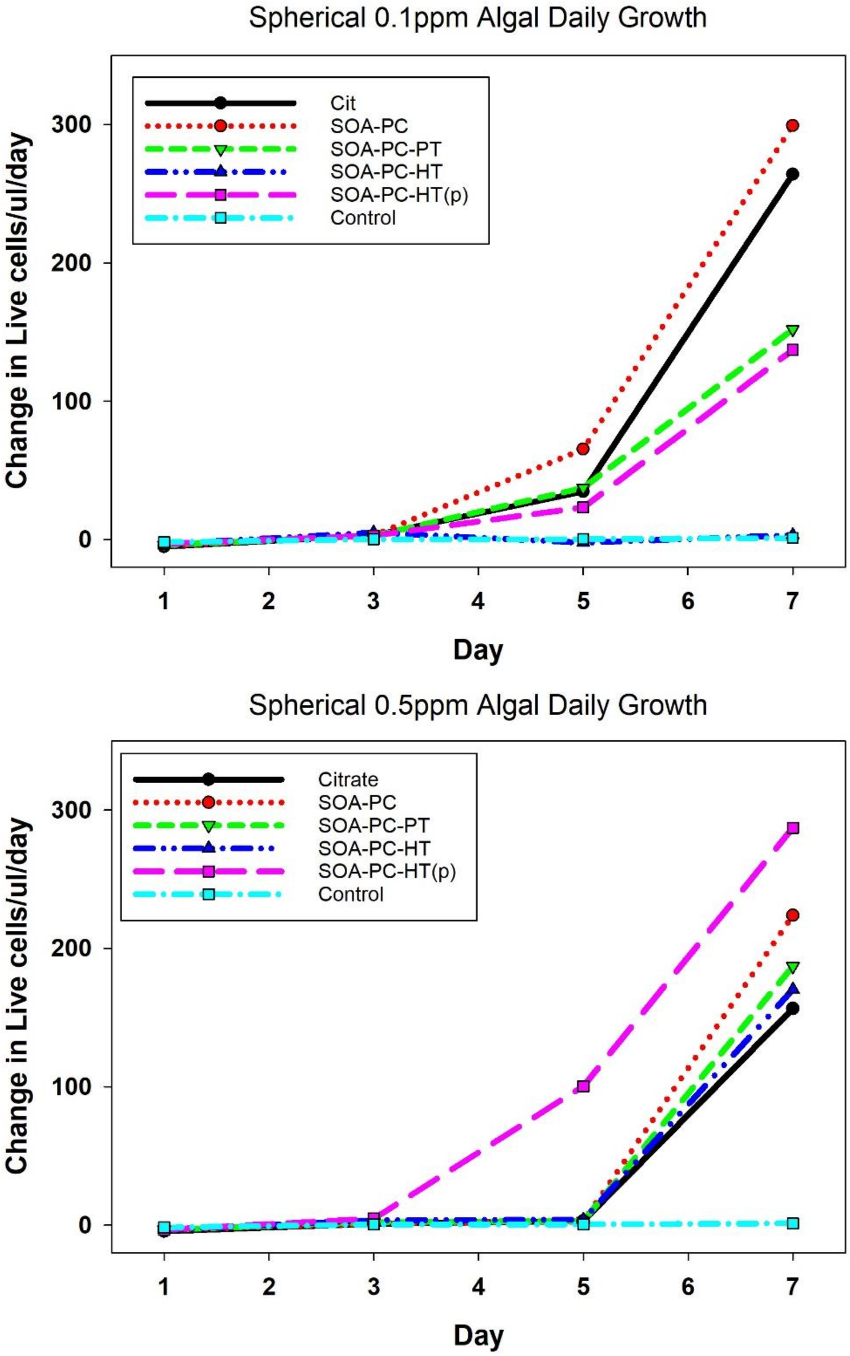

Figure S5: Change in the number of live algal cells following exposure to spherical AgNPs at 0.1 and 0.5 mg Ag/L over the 7-day experimental period.

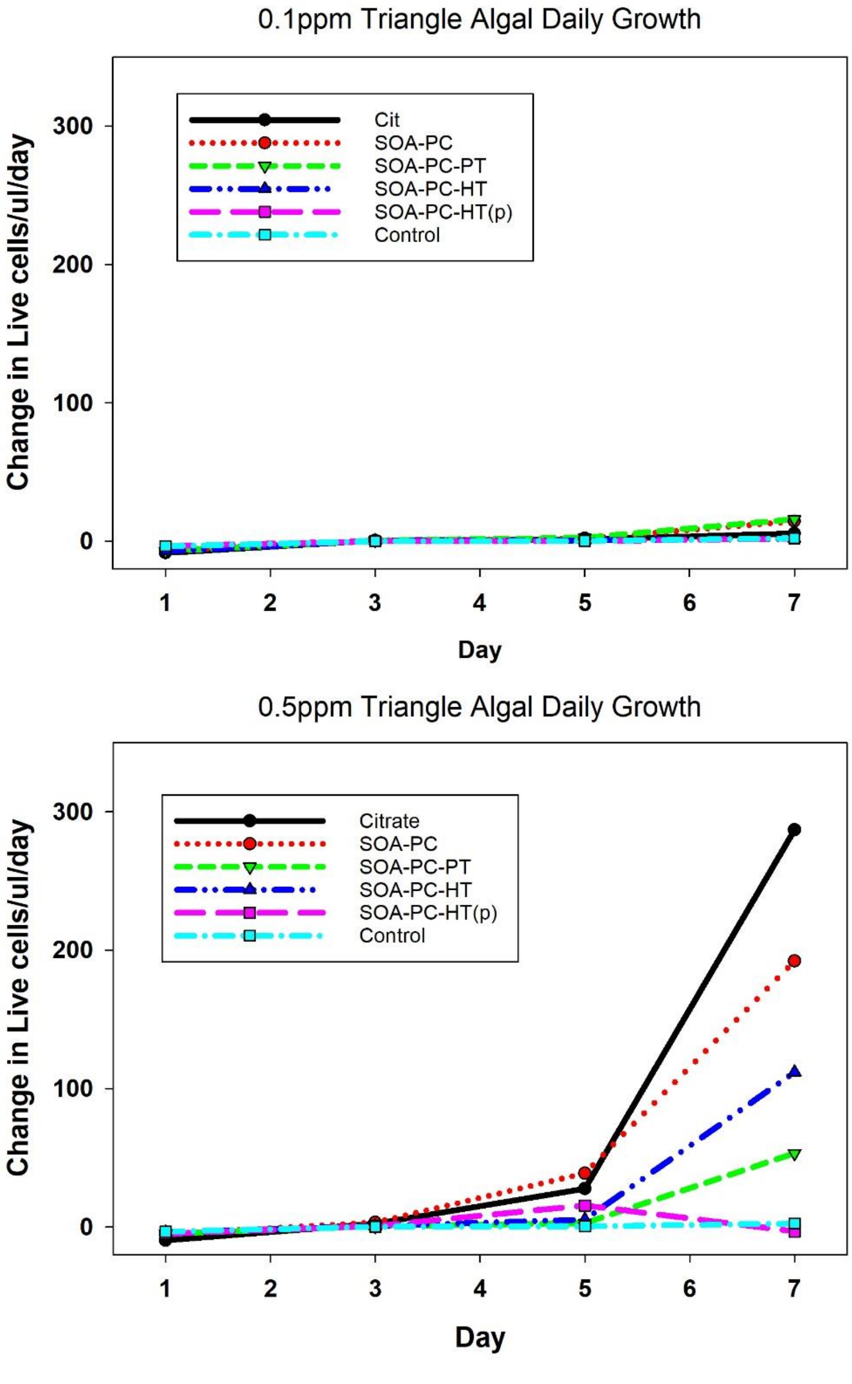

Figure S6: Change in the number of live algal cells following exposure to triangular AgNPs at 0.1 and 0.5 mg Ag/L over the 7-day experimental period.

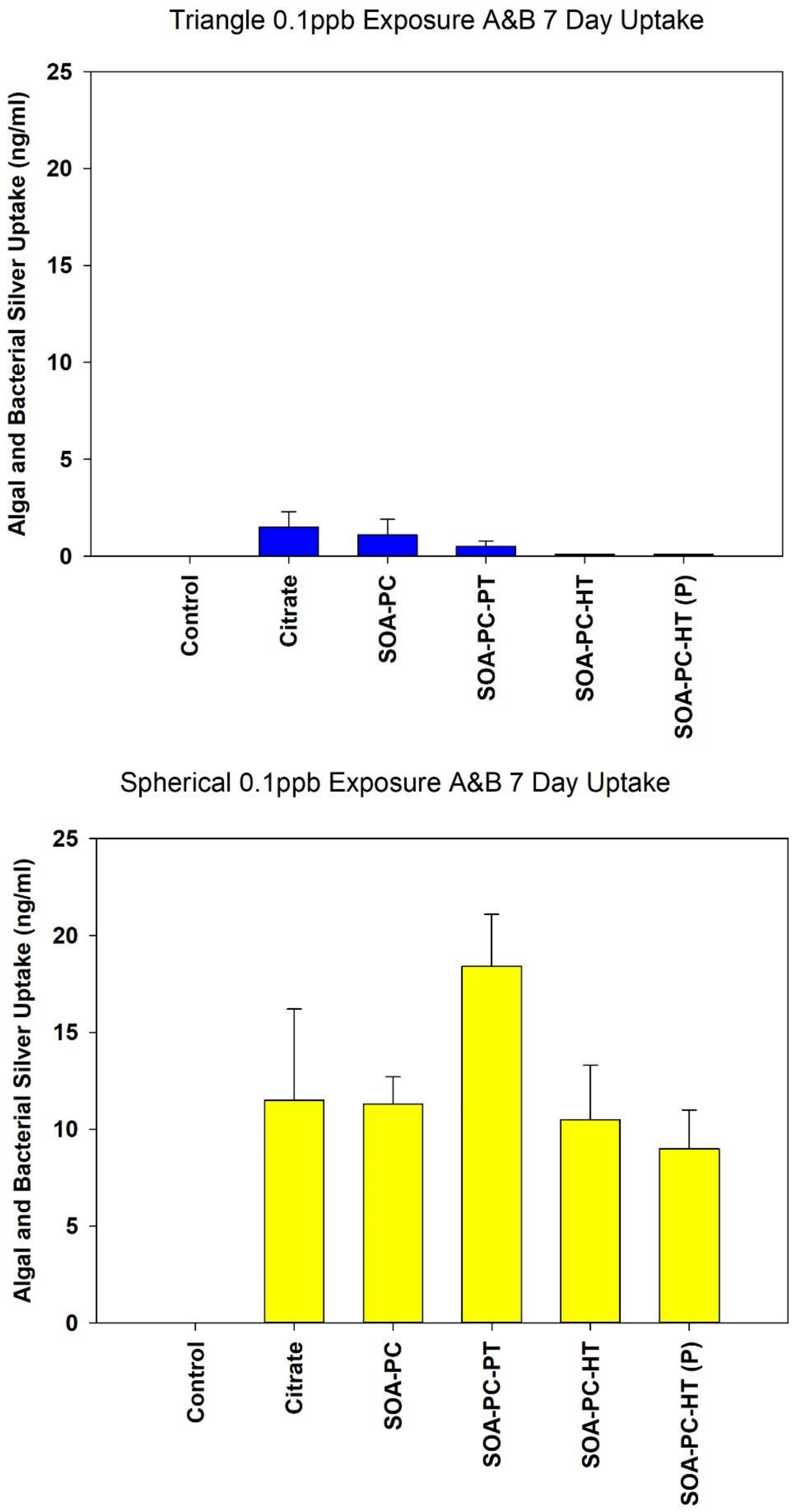

Figure S7: Silver uptake in algal and bacterial cells following 7 day exposure to 0.1 mg Ag/L of lipid coated triangular and spherical nanoparticles with varying surface chemistries.

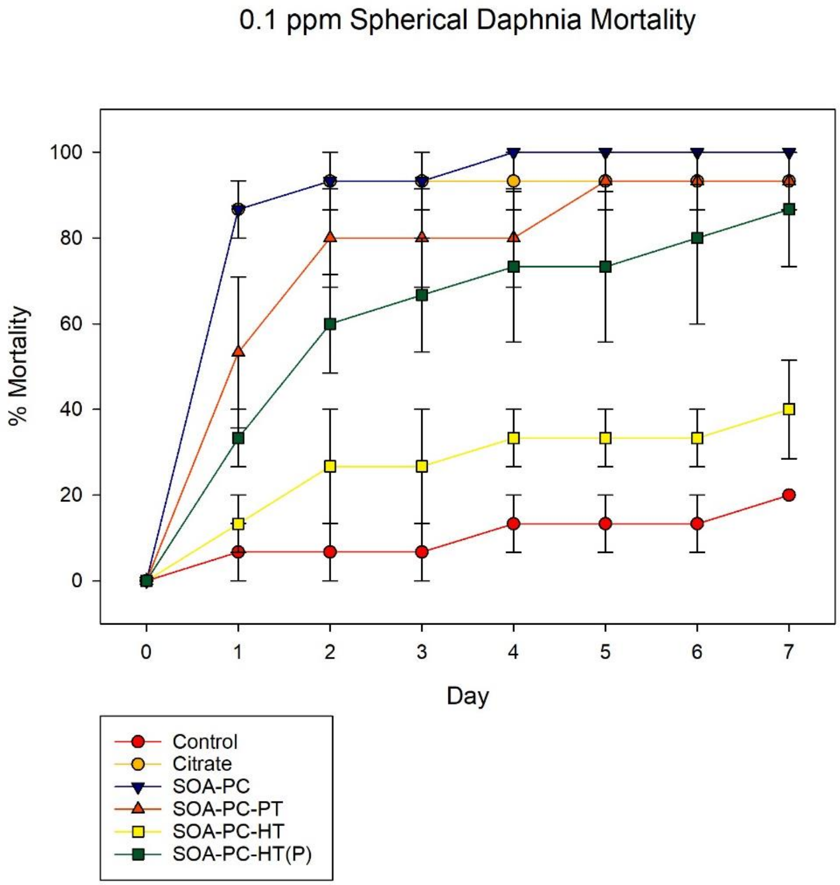

Figure S8: Daily mortality rate (%) for daphnids exposed to AgNSs at 0.1 mg Ag/L over the 7-day experimental period.

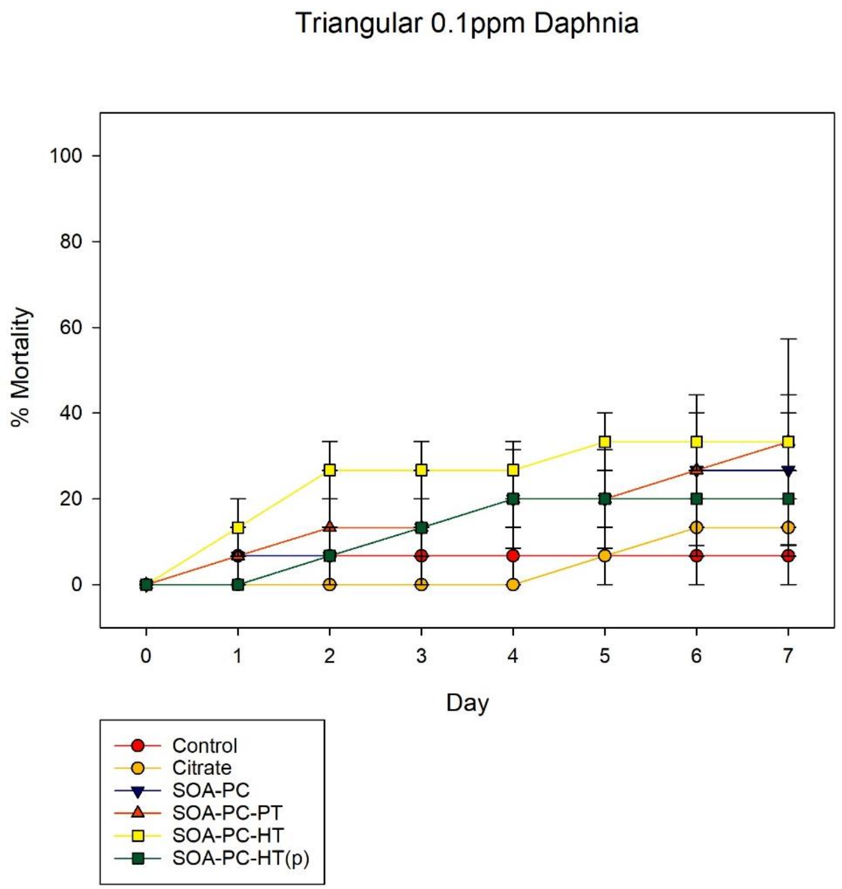

Figure S9: Daily mortality rate (%) for daphnids exposed to AgNPLs at 0.1 mg Ag/L over the 7-day experimental period.

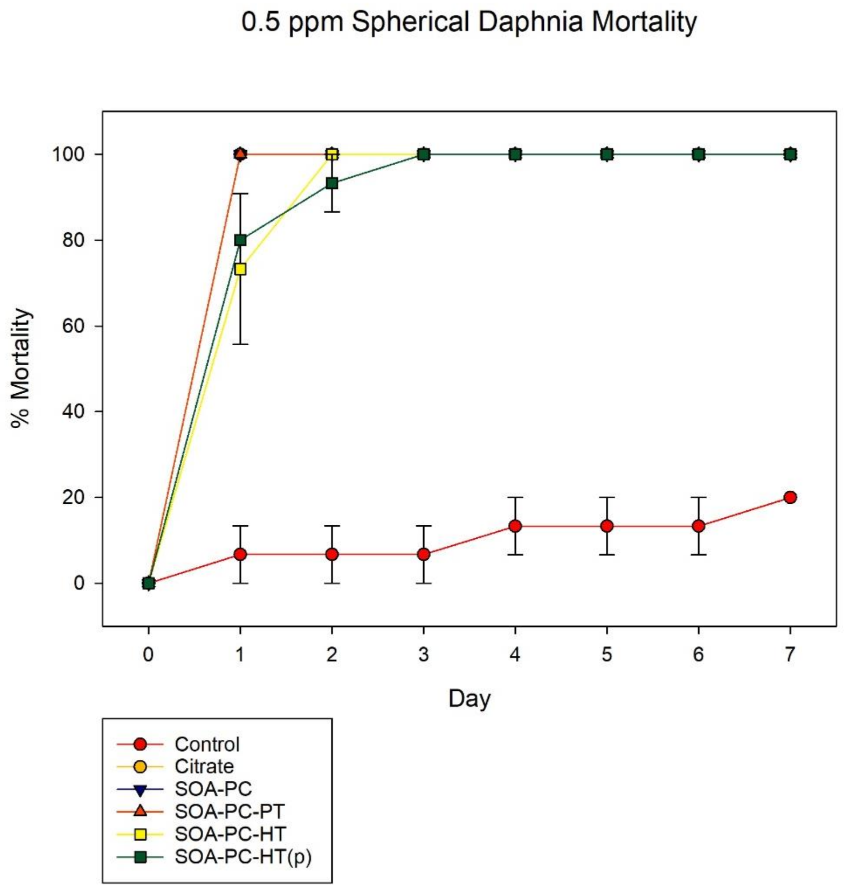

Figure S10: Daily mortality rate (%) for daphnids exposed to AgNSs at 0.5 mg Ag/L over the 7-day experimental period.

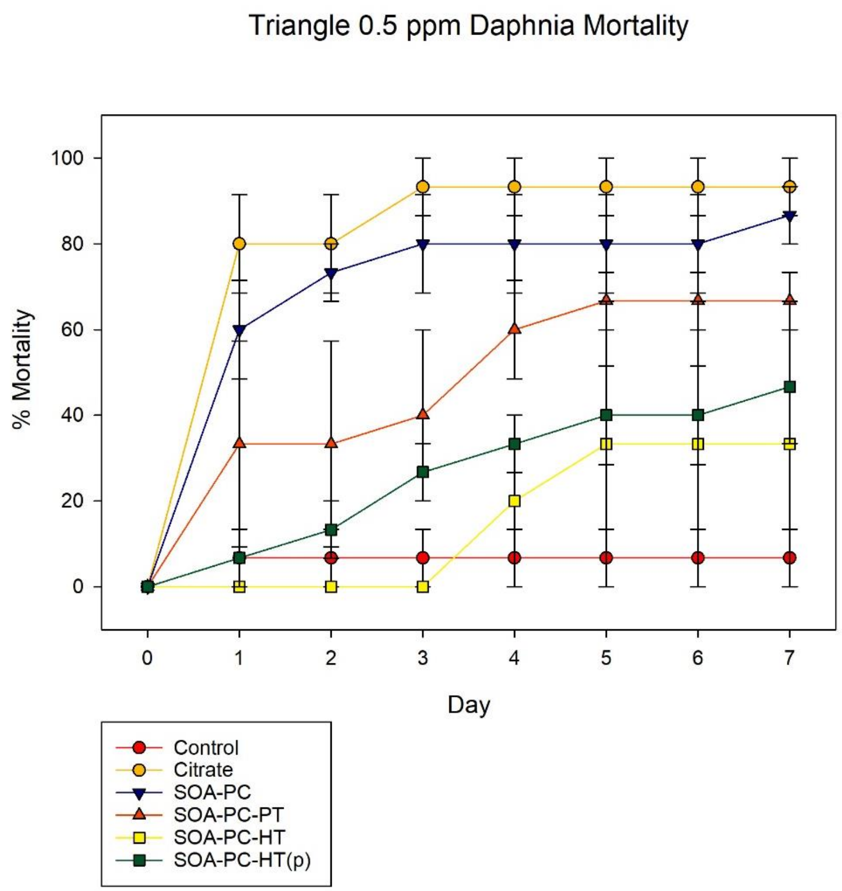

Figure S11: Daily mortality rate (%) for daphnids exposed to AgNPLs at 0.5 mg Ag/L over the 7-day experimental period.

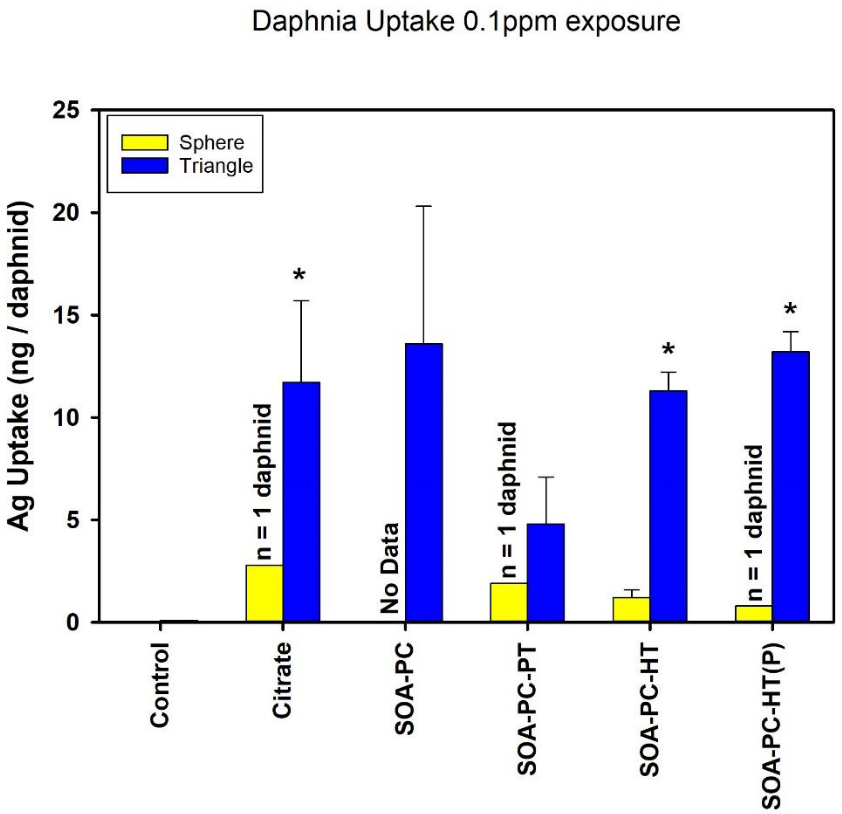

Figure S12: Silver uptake by *Daphnia* surviving the 7-day exposure as determined by ICP-MS. Asterisk indicates significance relative to control daphnid silver content.

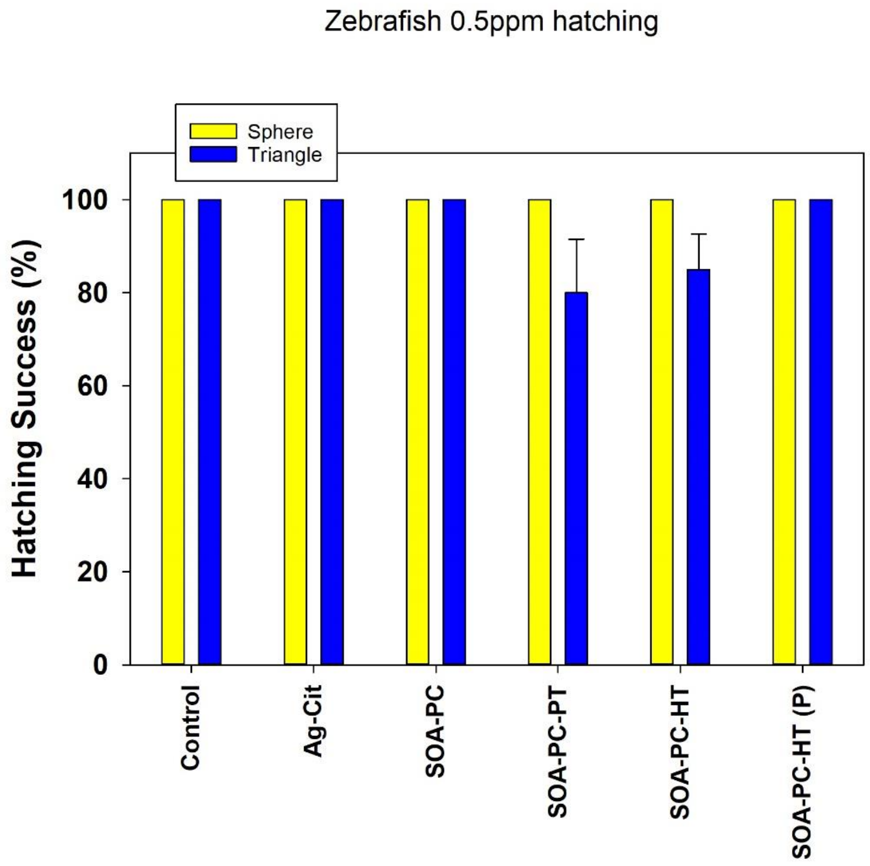

Figure S13: Hatching rate for zebrafish embryos exposed to spherical or triangular AgNPs at 0.5 mg Ag/L over the 7-day experimental period.
